# Template activating factor-I epigenetically regulates the *TERT* transcription in human cancer cells

**DOI:** 10.1038/s41598-021-97009-9

**Published:** 2021-09-06

**Authors:** Kohsuke Kato, Atsushi Kawaguchi, Kyosuke Nagata

**Affiliations:** 1 Department of Infection Biology, Faculty of Medicine, University of Tsukuba, 1-1-1 Tennodai, Tsukuba, 305-8575 Japan; 2 Transborder Medical Research Center, University of Tsukuba, Tsukuba, Japan; 3 Microbiology Research Center for Sustainability, University of Tsukuba, Tsukuba, Japan; 4 Faculty of Medicine, University of Tsukuba, Tsukuba, Japan

**Keywords:** Cancer, Cell biology, Molecular biology

## Abstract

Telomere, the terminus of linear chromosome in eukaryotes, is composed of specific repeat DNA which is mainly synthesized by a protein complex called telomerase. The maintenance of telomere DNA is important for unlimited proliferative capacity of cancer cells. The telomerase activity is controlled by the expression level of *telomerase reverse transcriptase* (*TERT*), a catalytic unit of telomerase, in some species including human. Therefore, to reveal the regulatory mechanisms of the transcription of *TERT* gene is important for understanding the tumor development. We found that template activating factor-I (TAF-I), a multifunctional nuclear protein, is involved in the transcriptional activation of *TERT* for the maintenance of telomere DNA in HeLa cells. TAF-I maintains the histone H3 modifications involved in transcriptional activation and hypomethylated cytosines in CpG dinucleotides around the transcription start site (TSS) in the *TERT* gene locus. Collectively, TAF-I is involved in the maintenance of telomere DNA through the regulation of *TERT* transcription, then consequently the occurrence and/or recurrence of cancer cells.

## Introduction

Telomeres are located at the ends of chromosome and have specific repetitive DNA sequence in eukaryotes. Because DNA polymerase cannot synthesize 5′-terminal regions, telomere DNA is gradually shortened every DNA replication in most terminally differentiated human cells (end replication problem)^1^. The shortened telomere DNAs are recognized by cellular DNA damage sensor proteins, then the DNA damage-signaling pathways induce the irreversible cell cycle-arrest through the activation of several cell cycle regulators such as p53. Thus, after repeating cell divisions, terminally differentiated cells usually enter the cell cycle-arrested state, called senescence, due to the limited dividing capacity^2^. This suggests that unlimited telomere DNA synthesis is essential to acquire immortality which consequently takes a chance to proceed the cancer.

The telomere DNA is synthesized by telomerase, a ribonucleoprotein complex consisting of a catalytic subunit telomerase reverse transcriptase (TERT), a template RNA telomerase RNA component (*TERC*), and several other proteins^3^. Telomerase is usually activated in stem cells and cancer cells. The expression level of TERT is highly regulated at both transcriptional and post-transcriptional level. The transcription from the *TERT* promoter is a primary determinant of the telomerase activity depending on cell types and cell states. As summarized in a review by Gaspar et al., telomerase reactivation is observed in approximately 90% of human cancer cells through the upregulation of *TERT* transcription^4^. A variety of transcription factors regulate the *TERT* transcription. Sp1 and c-Myc function as major transcriptional activators of *TERT* through their bindings on the *TERT* promoter region^5^. In contrast, several transcription factors such as CTCF and WT1 negatively regulate the transcription of *TERT*^6,7^*.* Several nucleotide mutations are frequently introduced in the *TERT* promoter region and implicated in telomerase reactivation in cancer cells by the de novo binding sites for ETS family transcription factors such as GABP^8–10^. In addition to such somatic mutations, amplification of *TERT* gene^11,12^ and rearrangement of *TERT* locus^13,14^ directing its transcriptional activation are also reported during cancer development. In addition to genetic mechanism, the transcription of *TERT* is also regulated by epigenetic mechanisms. *TERT* gene contains CpG islands located at the region from 838 bp upstream of the first AUG codon to the end of exon 2^15^. It is well known that 5-methyl-cytosine (5mC) in CpG dinucleotides functions as a pivotal epigenetic mark in gene silencing. In general, the hypomethylated status around the TSS in the *TERT* promoter is required for its transcriptional activation^16^. In contrast, as summarized in a review by Lee et al., many previous studies reveal that the hypermethylation in *TERT* locus is correlated with high expression level of telomerase in a variety of cancer cells^17^. In particular, a 433 bp-long genomic region including 52 CpG sites located upstream of the core promoter in *TERT* locus, called the TERT hypermethylated oncological region (THOR), is highly methylated and involved in the cancer-associated transcription of the *TERT*^18^. The hypermethylation of THOR promotes the *TERT* transcription by inhibiting the binding of transcription repressors such as CTCF and WT1^17^. Not only DNA methylation, but also several histone modifications involved in transcriptional regulation are also important epigenetic marks to regulate the *TERT* transcription. Acetylation of histone H3 K9 and K14 (K9K14ac)^19,20^ and tri-methylation of histone H3 K4 (K4me3)^19,21^ promotes the transcriptional activation of *TERT* through the formation of open chromatin structure. Upon transcriptional repression, tri-methylations of histone H3 K9 (K9me3)^20^ or K27 (K27me3)^15,21^ are introduced in the *TERT* gene locus to form closed chromatin structures. However, the detailed mechanism how the *TERT* gene activity is epigenetically regulated remains unclear.

Template activating factor-I (TAF-I) was originally identified as a host factor that activates adenovirus DNA replication and transcription through the remodeling of chromatin-like viral genome DNA–protein complexes^22^. Two subtypes of TAF-I, TAF-Iα and TAF-Iβ, are expressed from the *TAF-I* gene locus using two alternative promoters^23^. TAF-I has a histone chaperone activity against histone H3 and H1 in vitro^24–27^. In particular, we found that TAF-I is associated with several histone H1 variants and regulates its chromatin-binding dynamics in the nucleus^26^. TAF-I is involved in transcriptional regulation of interferon-stimulated genes (ISGs) through its histone H1 chaperone activity^28^. TAF-I also regulates epigenetic marks related to transcription without the histone chaperone activity. TAF-I inhibits the acetylation of histone H3 as a subunit of inhibitor of histone acetyltransferase (INHAT) complex^29,30^. Furthermore, it is reported that TAF-I indirectly regulates the level of DNA methylation through upregulating the expression of ten–eleven translocation 1 (TET1), a hydroxylation enzyme of methylated cytosine, for the DNA demethylation^31^. However, how each gene activity individually controlled by TAF-I through epigenetic mechanisms is still unclear.

Here, we found that TAF-I maintains the telomere integrity through the epigenetic regulation of the *TERT* gene transcription in human cancer cells. The transcription of *TERT* was downregulated by knock down (KD) of TAF-I. Histone H3 K9K14ac, K4me3, and DNA methylation around the TSS in *TERT* promoter are regulated by TAF-I. Collectively, we proposed that TAF-I is involved in the occurrence and/or recurrence of cancer cells by telomerase reactivation through the epigenetic mechanisms.

## Results

### Expression level of *TERT* mRNA, telomerase activity, and telomere DNA length are impaired in TAF-I KD HeLa cells

**Figure 1:**
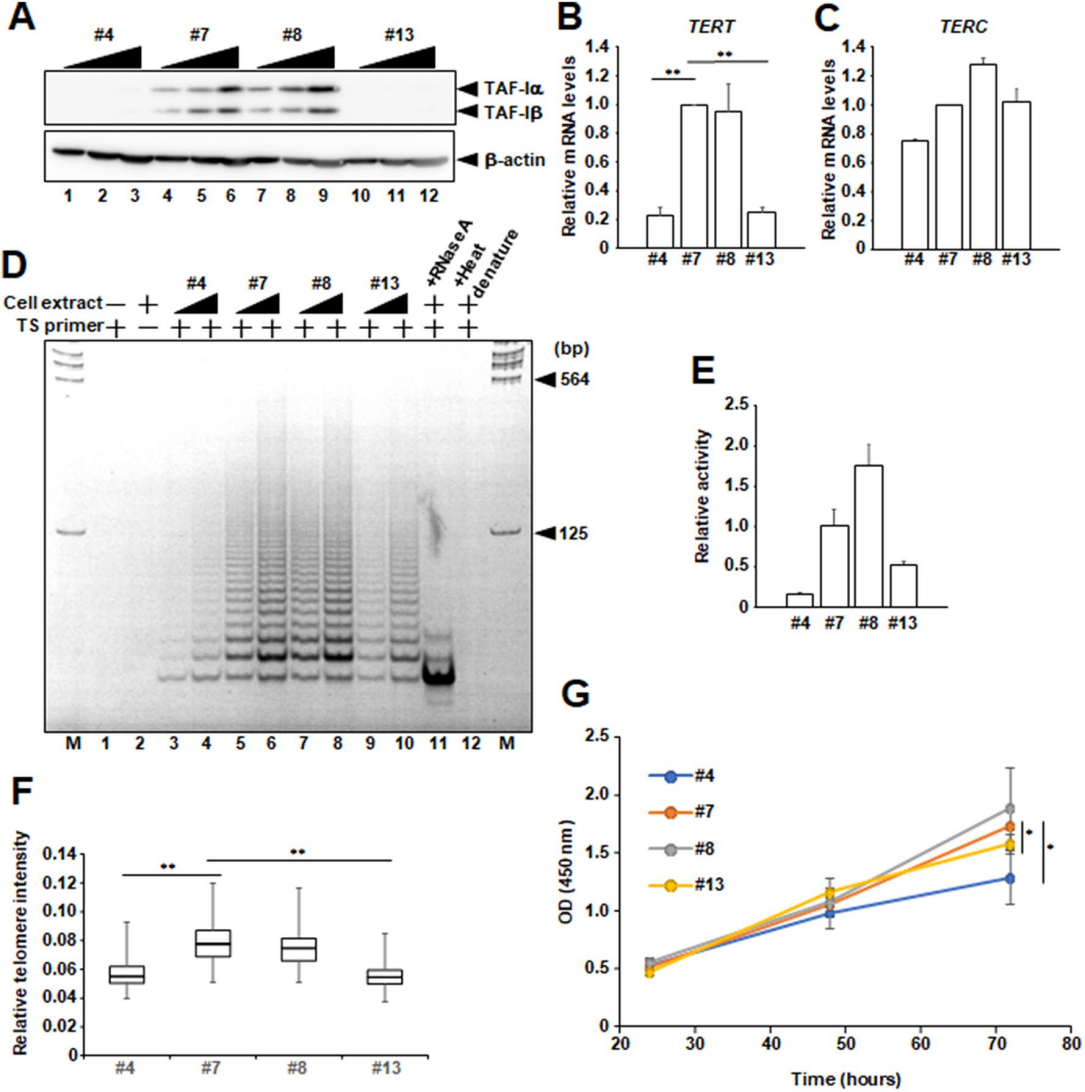
The *TERT* expression and telomerase activity is downregulated in TAF-I KD HeLa cell lines. (**A**) TAF-Iα and TAF-Iβ expression levels were examined by western blotting analyses. Total cell extracts prepared from WT (#7: lanes 4–6, and #8: lanes 7–9) and TAF-I KD (#4: lanes 1–3, and #13: lanes 10–12) HeLa cell lines were separated on 10% SDS-PAGE followed by western blotting analyses using anti-TAF-Iα/β and anti-β-actin antibodies. Five, ten, and twenty µg of total protein prepared from each cell line was loaded. (**B**,**C**) The expression level of *TERT* mRNA and *TERC* RNA was examined by Q-RT-PCR analyses in each HeLa cell lines. The 18S rRNA was used for normalization as an internal control. Values represent the mean ± SD (n = 3). **P < 0.01 relative to #7. (**D**) Telomerase activity was examined by TRAP assay. Each cell extract derived from 5.0 × 10^2^ cells (lanes 3, 5, 7, and 9) and 2.5 × 10^3^ cells (lanes 4, 6, 8, and 10) was incubated as described in “Materials and methods”. Reactions were done without cell extract (lane 1) or TS primer (lane 2) as negative controls. Cell extract pre-treated with RNase A (lane 11) or heat-denatured (lane 12) was used to confirm the telomerase-dependency on reactions. (**E**) Q-TRAP assay. Each cell extract derived from 1.0 × 10^2^ cells was incubated in reaction mix and subjected to Q-PCR analyses as described in “Materials and methods”. Values represent the mean ± SD (n = 3). (**F**) Relative telomere intensity of WT and TAF-I KD cells. A value of total telomere intensity in one cell nucleus was normalized by dividing it with a value of total DAPI signal in same cell nucleus, then plotted as a box plot. At least, images of more than 130 cells were taken and analyzed in each cell clone. **P < 0.01 relative to #7. (**G**) Cell growth assay in HeLa cell lines. The horizontal line indicates time after plating cells, and the vertical line indicates the absorbance at 450 nm after adding WST-8 reagents. Values represent the mean ± SD (n = 3). *P < 0.05 relative to #7.

From previous our microarray analyses, we have identified *TERT* as one of downregulated genes at mRNA level in shRNA-mediated TAF-I KD HeLa cell lines^32^. To confirm this, we examined the amount of *TERT* mRNA in TAF-I KD HeLa cell lines^33^ by quantitative RT-PCR (Q-RT-PCR) analyses. We used two HeLa cell lines (clone #7 and #8) expressing both TAF-Iα and TAF-Iβ at the similar levels of intact HeLa cells, defined as WT cells, and two other cell lines (clone #4 and #13), in which TAF-I expression is strongly reduced compared to WT cells, defined as TAF-I KD cells (Fig. 1A, Supplementary Fig. 1A–E). The amount of the *TERT* mRNA in both clone #4 and #13 TAF-I KD cells was reduced to approximately 20% of WT cells (Fig. 1B). We also examined the amount of *TERC* RNA in both WT and TAF-I KD cells (Fig. 1C). Although we found that the amount of *TERC* RNA in clone #4 cells was slightly reduced compared to WT cells, the reduction of *TERT* mRNA was higher than that of *TERC* RNA. Next, we examined the enzymatic activity of telomerase in TAF-I KD cells by telomeric repeat amplification protocol (TRAP) assays. We performed TRAP assays with appropriate PCR cycles to prevent a saturation of PCR amplicons (Supplementary Figs. 2A and 8A). The efficiently amplified PCR products was detected in the cell lysates prepared from WT cells (Fig. 1D, lanes 5–8, and Supplementary Fig. 8B), and the amplified products were not observed in the RNase A-treated or heat-denatured cell lysates (Fig. 1D, lanes 11 and 12), indicating that the amplification of PCR products is dependent on the telomerase activity. In contrast, the amount of PCR products decreased in the cell lysates prepared from TAF-I KD cells compared with that of WT cells (Fig. 1D, lanes 3–4 and lanes 9–10), suggesting that the telomerase activity in TAF-I KD cells are impaired possibly due to the reduction of the *TERT* mRNA. These results suggest that TAF-I is involved in the maintenance of the telomerase activity in HeLa cells through upregulating *TERT* expression. Notably, the amount of β-actin in the cell lysates was not changed between each cell line, suggesting that the cell lysates were prepared from each cell line to similar extents (Supplementary Figs. 1F,G, 2B). For more quantitative analyses of telomerase activity, we also performed Q-PCR-based TRAP (qTRAP) assay. The telomerase activity in TAF-I KD cells is also lower than that in WT cells similarly observed in Fig. 1D (Fig. 1E). Furthermore, we examined the length of telomere repeat DNA in TAF-I KD cells by telomere-FISH assays^34^. The relative intensity of telomere repeat DNA in TAF-I KD cells was lower than that of WT cells (Fig. 1F, Supplementary Figs. 3, and 4A–D). We also performed a TRF assay to evaluate the length of telomere repeat DNA. In both WT cells, we detected approximately 14 kbp-peaked TRFs (Supplementary Figs. 5A,B, 8C,D). In TAF-I KD #4 cells, approximately 4.5 kbp-peaked TRF was clearly detected (Supplementary Fig. 5A,B). In contrast to #4 cells, we detected relatively longer TRF DNA in TAF-I KD #13 cells, but it was actually shortened compared to that in WT cells (Supplementary Fig. 5A,B). These results suggest that telomere repeat DNA in TAF-I KD HeLa cells becomes relatively shorter than that in WT cells. Furthermore, we examined whether TAF-I KD affects cell growth of HeLa cell lines. We found that the cell growth of TAF-I KD cells is moderately retarded compared to that in control cells after 72 h post cell plating (Fig. 1G). It is possible that TAF-I KD affects cell growth through the regulation of *TERT* transcription in HeLa cells.

### The reduction of *TERT* expression in TAF-I KD cells is not due to the instability of *TERT* mRNA

**Figure 2:**
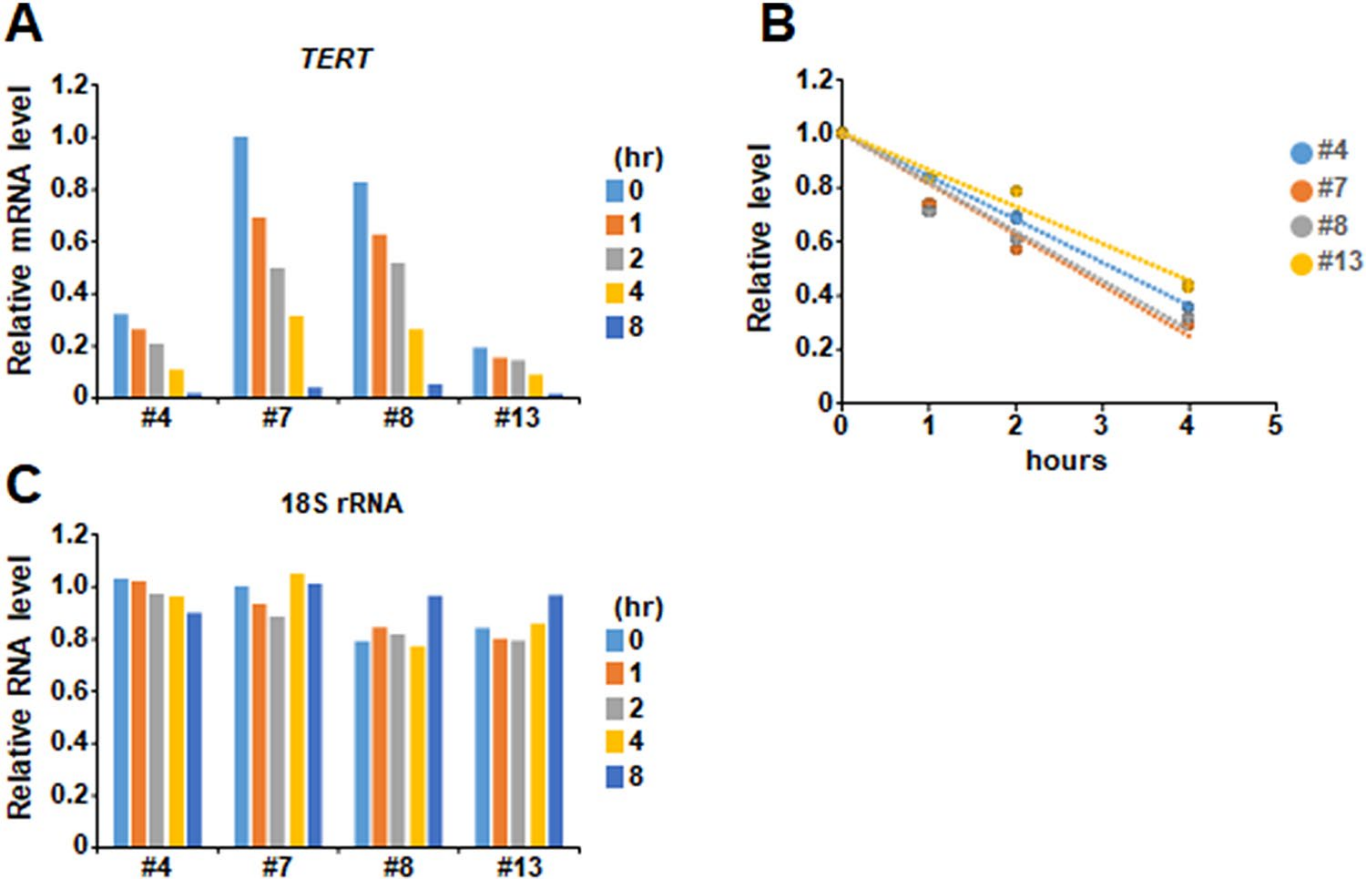
Stability of the *TERT* mRNA was not so different between WT and TAF-I KD cells. (**A**,**C**) The expression level of *TERT* mRNA in WT and TAF-I KD cells which were treated with actinomycin D was examined by Q-RT-PCR analyses. WT and TAF-I KD cells treated with actinomycin D for 1, 2, 4 and 8 h were collected and subjected to total RNA purification. Each cell just before treating with actinomycin D was also collected as a 0 h sample. Q-RT-PCR analyses were performed with each purified total RNA to examine the expression level of *TERT* mRNA and 18S rRNA. Relative expression level of *TERT* mRNA and 18S rRNA was plotted as indicated graphs (**A**,**C**), respectively. (**B**) The value of *TERT* mRNA was normalized by dividing it with that of 18S rRNA. Each relative value when the normalized value of 0 h sample was set to be 1.0 was represented.

The amount of *TERT* mRNA is regulated at both transcriptional and post-transcriptional levels. It is reported that the stability of *TERT* mRNA is upregulated by poly(C) binding proteins through its binding to the 3′-UTR of *TERT* mRNA^35^. To clarify whether the reduction of *TERT* mRNA in TAF-I KD cells is caused by post-transcriptional regulation, we examined the stability of *TERT* mRNA in WT and TAF-I KD cells. At 1, 2, 4, and 8 h post treatment of actinomycin D, which is a potent inhibitor of transcription, the amount of *TERT* mRNA was examined by Q-RT-PCR. The amount of *TERT* mRNA in each cell line gradually decreased to a similar extent in a time-dependent manner by adding actinomycin D (Fig. 2A,B), while the amount of 18S rRNA was unchanged (Fig. 2C). This suggests that the stability of *TERT* mRNA is not impaired by TAF-I KD.

### Transcription from transiently transfected *TERT* promoter-driven reporter gene is not downregulated in TAF-I KD cells

**Figure 3:**
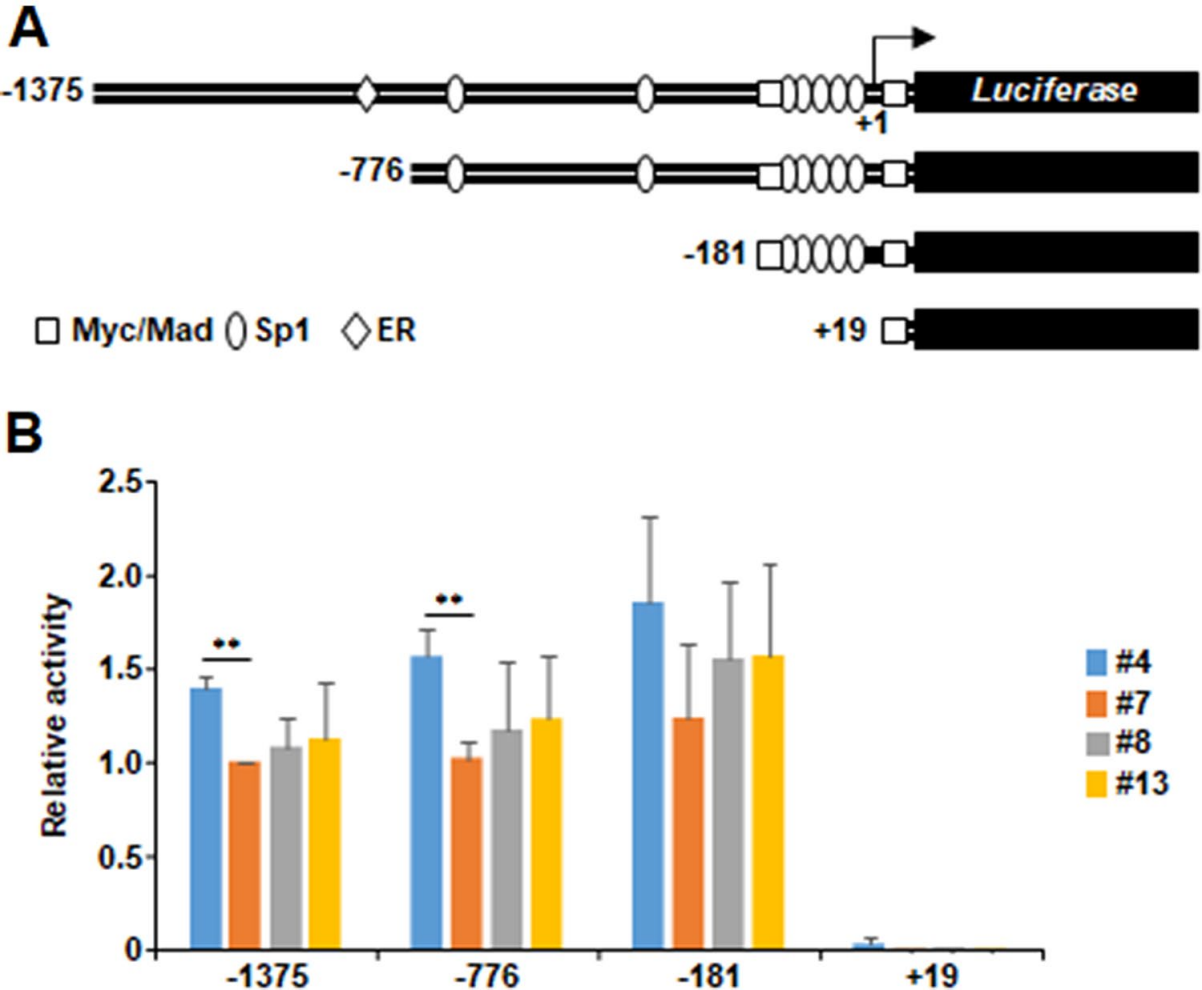
Transcription from transiently transfected pTERT-Luc reporter plasmids could not reconstitute the downregulation of endogenous *TERT* transcription by TAF-I KD. (**A**) Schematic diagrams of pTERT-Luc reporter genes. The upstream and downstream nucleotide positions from transcription start site (+ 1) of each pTERT-Luc plasmid are shown. Binding sites for major transcription factors such as Myc/Mad, Sp1, and ER are represented. (**B**) Results of luciferase assay. Each pTERT-Luc reporter plasmid together with pEF1α-RLuc control plasmid were transfected into WT and TAF-I KD cells. *Renilla* luciferase was used as an internal control. After 48 h post transfection, cells were collected, and cell extract was prepared and subjected to measure both firefly and *renilla* luciferase activities. Each value of firefly luciferase activity was normalized by dividing it with that of *renilla* luciferase. Each relative value when the normalized value of pTERT-1375-Luc-transfected clone #7 cells was set to be 1.0 was represented. Values represent the mean ± SD (n = 3). **P < 0.01 relative to #7.

TAF-I is involved in the transcriptional regulation of a variety of genes through multiple mechanisms. TAF-I directly regulates the DNA binding activity of several transcription factors such as Sp1 and regulates the transcription of their downstream genes^36,37^. In addition, it was reported that TAF-Iβ promotes the stability of c-Myc through the inhibition of PP2A-mediated dephosphorylation^38^. Therefore, there is a possibility that TAF-I directly regulates the transcription of *TERT* through Sp1 and/or c-Myc. We performed reporter gene assays using plasmids harboring human *TERT* promoter-driven firefly *luciferase* (Luc) gene (pTERT-Luc). pTERT-Luc plasmids having different length of human *TERT* promoter^39^ were examined (Fig. 3A). Both Sp1 and c-Myc are critical activators of the *TERT* promoter on these constructs^39^. The luciferase activity in TAF-I KD clone #13 cells transfected with pTERT-Luc-1375, -776, and -181 was comparable to that of WT cells, respectively (Fig. 3B). In addition, the luciferase activity in TAF-I KD clone #4 cells transfected with pTERT-Luc-1375, and -776 was not reduced, rather showed a little higher activity than that of WT clone #7 cells, respectively (Fig. 3B). Note that a plasmid lacking the transcription start site (pTERT-Luc + 19) did not show any luciferase activity, indicating that the *TERT* promoter on reporter plasmids is properly activated in this assay condition (Fig. 3B). Thus, it is possible that the reduction of the *TERT* promoter activity by TAF-I KD is not reconstructed on transiently transfected reporter plasmids in HeLa cells. It is suggested that transiently transfected plasmid DNA does not form a well-organized chromatin structures because of irregular histone binding^40^. Considering these facts, we assumed that TAF-I is involved in the transcriptional activation of *TERT* on chromatinized DNA through the epigenetic mechanisms.

### The methylation level of CpG dinucleotides around TSS of the *TERT* is increased by TAF-I KD

**Figure 4:**
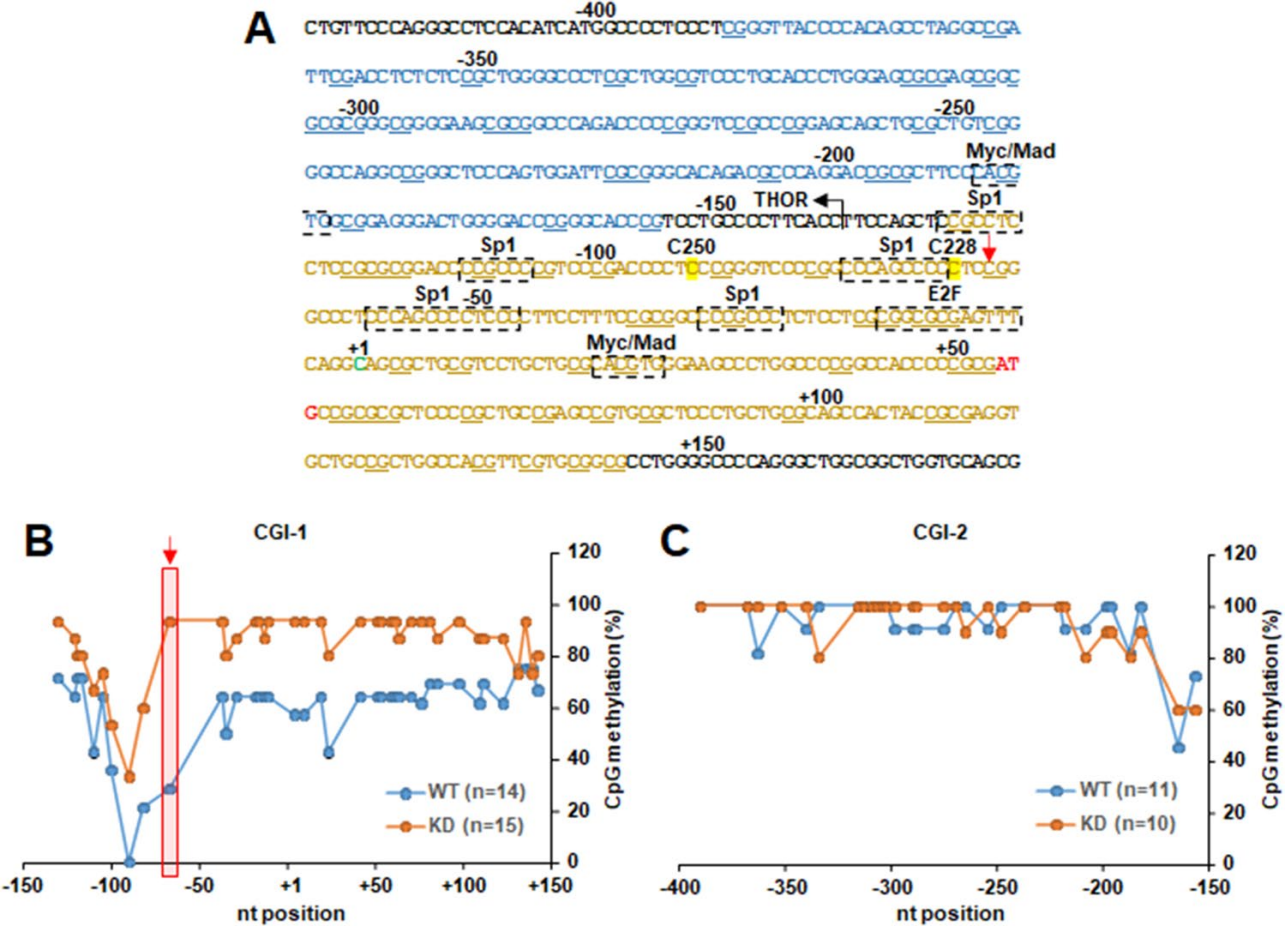
The level of CpG dinucleotides methylation around the *TERT* TSS was higher in TAF-I KD cells compared to that of WT cells. (**A**) The DNA structure in human *TERT* locus analyzed in bisulfite sequencing analyses. DNA sequence corresponding to the region from − 424 to + 176 nucleotide positions of TSS in human *TERT* locus is represented. CpG island region-1 (CGI-1) and CGI-2 are shown by yellow and blue letters, respectively. All analyzed CpG dinucleotides are indicated by under bars. The nucleotide positions of transcription site (+ 1) and a translation initiation codon (first ATG) of the *TERT* are shown by green and red letters, respectively. The nucleotide positions of C228T and C250T mutations are highlighted with yellow marker. Binding sites for major transcription factors such as Myc/Mad, Sp1, and E2F are indicated by dashed lined boxes. The start site of THOR is indicated by a bended arrow. The position of cytosine showing the most marked difference of methylation level between WT and TAF-I KD cells is indicated by a red arrow. (**B**,**C**) Bisulfite sequencing analyses. The methylation level of each CpG dinucleotide in CGI-1 (**B**) and CGI-2 (**C**) of the *TERT* locus was compared between WT and TAF-I KD cells. The horizontal line indicates nucleotide positions from the *TERT* TSS, and the vertical line indicates the percentage of CpG methylation. In (**B**), the position of cytosine showing the most marked difference of methylation level between WT and TAF-I KD cells is indicated by a red arrow and a shadowed box (please note a red arrow in (**A**), too). Total numbers of DNA clone used for sequencing were 14 (clone #7: 8, and #8: 6) for WT and 15 (clone #4: 9, and #13: 6) for TAF-I KD in CGI-1 (**B**), and 11 (clone #7: 7, and #8: 4) for WT and 10 (clone #4: 6, and #13: 4) for TAF-I KD in CGI-2 (**C**), respectively.

The cytosine methylation in CpG dinucleotides generally represses transcription by recruiting transcriptional repressors or co-repressors onto promoter regions including the *TERT* gene. However, DNA methylations in THOR and gene body region in the *TERT* gene locus are involved in its transcriptional activation by suppressing the binding of several transcription repressors including CTCF. Thus, it is suggesting that the CpG methylation-mediated gene regulation has more complicated molecular functions in the regulation of *TERT* transcription and the cancer progression^16,18^. We next tested the cytosine methylation of CpG islands (CGIs) located at the *TERT* promoter in TAF-I KD cells by bisulfite sequencing analyses. The *TERT* promoter region contains two CGIs between nucleotide positions − 130 to + 143 (CGI-1), and between nucleotide positions − 389 to − 155 (CGI-2) relative to the TSS (Fig. 4A)^20^. Note that CGI-2 is completely included in THOR^18^. In WT cells, the level of CpG methylations at nucleotide positions − 100 to − 50 within core promoter region of CGI-1 was lower than that of other regions including CGI-2 (Fig. 4B). It suggests that the low level of CpG methylation around the TSS is important for transcriptional activation of *TERT*. We found that the CpG methylation level around TSS in CGI-1 increased about 1.5- to 3-fold by TAF-I KD, although CGI-2 does not show significant differences between WT and TAF-I KD cells (Fig. 4B,C). These results suggest that TAF-I activates the *TERT* expression by decreasing the level of CpG methylation around the TSS, but not upstream THOR in the *TERT* locus. Previously, it was reported that over-expression of TAF-Iβ indirectly promotes the global CpG demethylation of genomic DNA through the upregulation of TET1 expression in HEK293 cells^31^. However, the *TET1* was poorly expressed in HeLa cells in contrast to other cancer cells (Supplementary Fig. 6A,B). Therefore, we also examined the level of 5-hydroxymethyl-cytosine (5hmC) at the CGI-1 in *TERT* by methylated DNA-immunoprecipitation (MeD-IP) assays. As a result, we could not observe the reduction of 5hmC in TAF-I KD cells compared to that in WT cells (Supplementary Fig. 7), suggesting that TAF-I-mediated maintenance of low level CpG methylation at CGI-1 in *TERT* is independent of the upregulation of TET1 expression.

**Figure 5:**
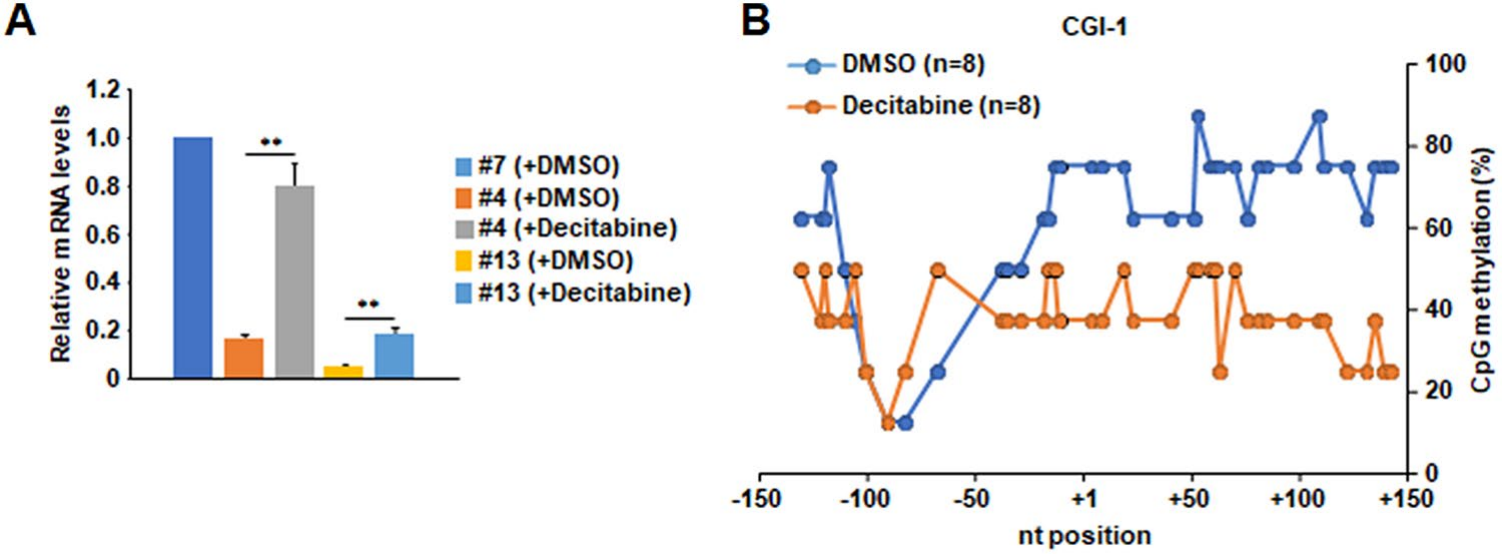
Decitabine-treatment upregulated *TERT* transcription through the reduction of CpG methylation at CGI-1 of *TERT* in TAF-I KD cells. (**A**) The expression level of *TERT* mRNA was examined in decitabine-treated HeLa cell lines. WT clone #7, TAF-I KD clone #4 and clone #13 cells were cultured in medium containing 0.003% DMSO (vehicle) or 30 µM decitabine for 4 days. Total RNA was isolated from each cell, and was subjected to Q-RT-PCR analyses to examine the expression level of *TERT* mRNA. The 18S rRNA was used for normalization as an internal control. Values represent the mean ± SD (n = 3). **P < 0.01 relative to each TAF-I KD cells treated with DMSO. (**B**) Bisulfite sequencing analyses. The methylation level of each CpG dinucleotide at CGI-1 of *TERT* was compared between DMSO or decitabine-treated TAF-I KD clone #4 cells. The horizontal line indicates nucleotide positions from the *TERT* TSS, and the vertical line indicates the percentage of CpG methylation.

To address whether the fluctuation of CpG methylation at CGI-1 is correlated with transcriptional activity of *TERT*, we performed Q-RT-PCR and bisulfite sequencing analyses in TAF-I KD cells treated with decitabine (5-aza-2′-deoxycytidine), a DNA methyltransferase inhibitor. The amount of *TERT* mRNA in decitabine-treated TAF-I KD cells was approximately fourfold increased compared to that in DMSO-treated cells (Fig. 5A). In parallel, the level of CpG methylation in decitabine treated-TAF-I KD #4 cells was broadly decreased to approximately one-half compared to that in DMSO-treated (Fig. 5B), suggesting that CpG methylation at CGI-1 of *TERT* regulates its transcriptional activity. Taken together, these results suggests that TAF-I is involved in transcriptional activation of *TERT* through the maintenance of lower CpG methylation around *TERT* TSS.

### The histone modifications involved in transcriptional activation were down-regulated on the *TERT* promoter by TAF-I KD

**Figure 6:**
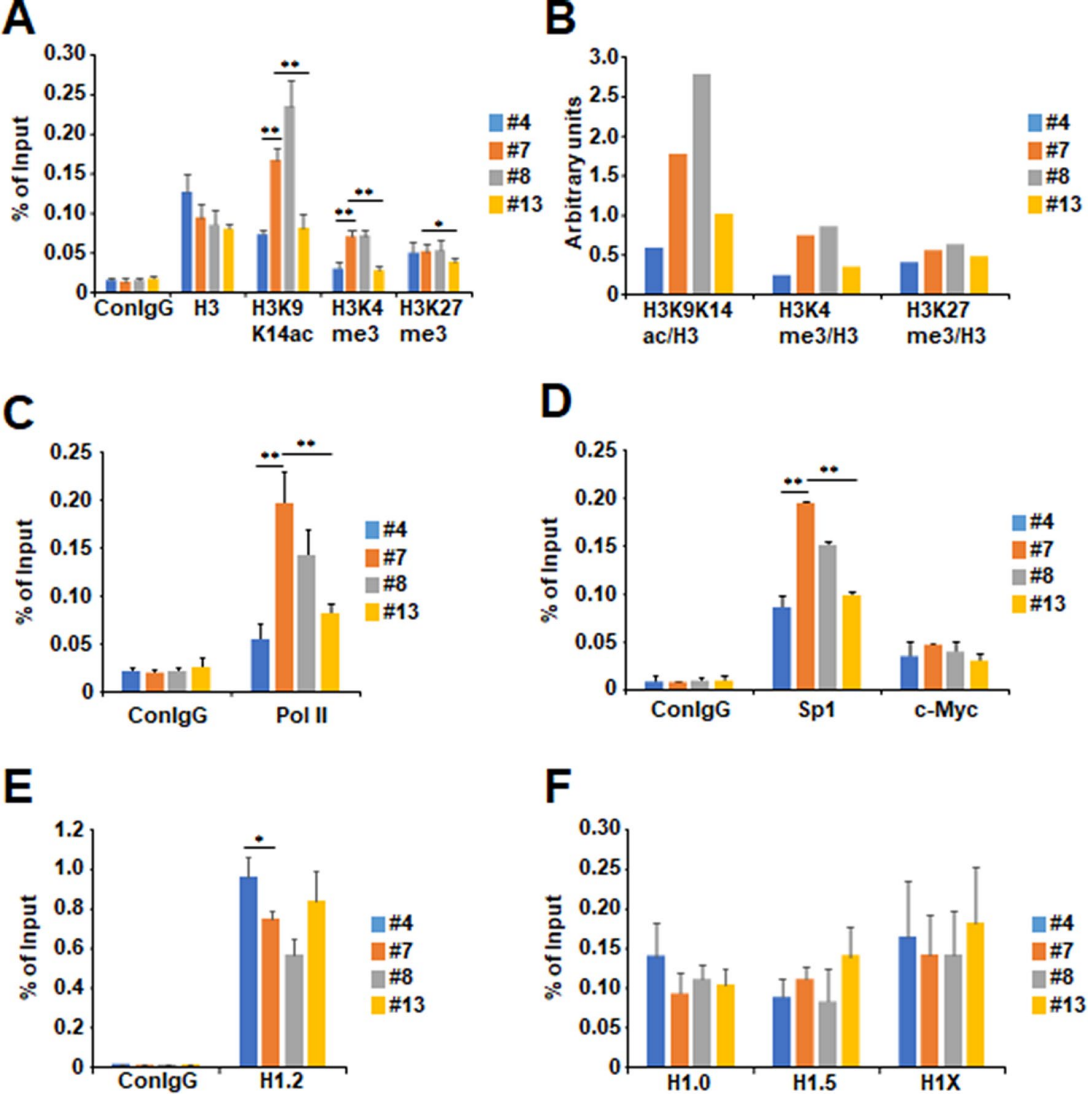
Histone modifications and Sp1 involved in transcriptional activation on *TERT* promoter were decreased in TAF-I KD HeLa cell lines independently of histone H1 amounts. ChIP assays were performed using WT and TAF-I KD HeLa cell lines. (**A**) The level of histone H3, H3 acetylated K9 and K14 (H3K9K14ac), H3 trimethylated K4 (H3K4me3), and H3 trimethylated K27 (H3K27me3) around the *TERT* TSS including core promoter was examined. Rabbit normal IgG was used as a control for monitoring background (ConIgG). Data were represented as % of input at vertical line. Values represent the mean ± SD (n = 3). **P < 0.01 and *P < 0.05 relative to #7. (**B**) The value of each histone H3 modification was normalized by that of histone H3. Arbitrarily units were represented at vertical line. (**C**,**D**) The level of rabbit normal IgG (conIgG), RNA polymerase II (pol II), Sp1, and c-Myc around the *TERT* TSS including core promoter was examined. Values represent the mean ± SD (n = 3). **P < 0.01 relative to #7. (**E**,**F**) The level of rabbit normal IgG, histone H1.2, H1.0, H1.5, and H1X around the *TERT* TSS including core promoter was examined. Due to the difference of scale of vertical line, data were separately represented for histone H1.2 (**C**) and other histone H1s (**D**). Values represent the mean ± SD (n = 3). *P < 0.05 relative to #7.

In general, CpG methylation and histone modifications cooperatively regulates the transcription through the several mechanistic interactions^41^. It is reported that histone H3 K9K14ac and K4me3 are involved in transcriptional activation of *TERT*, while histone H3 K9me3 and K27me3 oppositely repress it. We next examined the transcription-related histone modifications on the *TERT* promoter in TAF-I KD cells by chromatin immunoprecipitation (ChIP) assays. We measured the amount of histone H3 K9K14ac and K4me3 as markers for actively transcribed gene, and histone H3 K27me3 as a marker for transcriptionally silenced gene. Although the amount of histone H3 around the *TERT* TSS in TAF-I KD clone #4 cells was slightly increased, that in TAF-I KD clone #13 cells was not changed (Fig. 6A), suggesting that the amount of histone H3 is not involved in the downregulation of the *TERT* transcription in TAF-I KD cell lines. The level of histone H3 K9K14ac and K4me3 in both TAF-I KD cells was reduced to about 30 to 40% of that in WT cells (Fig. 6A,B). In contrast, histone H3 K27me3, a marker of transcriptionally silenced gene, was not dramatically changed by TAF-I KD (Fig. 6A,B). These results strongly suggest that TAF-I is involved in the transcriptional activation of *TERT* through the upregulation of histone modifications implicated in the transcriptional activation such as histone H3 K9K14ac and K4me3. In addition, we performed ChIP assay to examine the amount of RNA polymerase II (pol II), Sp1 and c-Myc to reveal the effect of TAF-I KD on transcription machineries. The amount of pol II in TAF-I KD cells was reduced to about 50% of that in WT cells (Fig. 6C). The amount of promoter-bound Sp1 but not c-Myc in TAF-I KD cells was also reduced to about 50 to 60% of that in WT cells (Fig. 6D). These results suggest that the reduction of chromatin-bound Sp1 through the changes of epigenetic marks is one of causes on TAF-I KD-dependent downregulation of *TERT* transcription.

Histone H1 is generally involved in transcriptional repression through the formation of highly packed chromatin structure^42^. We previously revealed that TAF-I is involved in the transcriptional repression through the deposition of histone H1.2 onto the promoter region of ISGs^28^. It is also reported that histone H1 suppresses the transcription through the inhibition of histone acetylation^43,44^ and the recruitment of DNA methyltransferase (DNMT) proteins^45^. ChIP assays were performed using antibodies against H1.2 and H1.5, which are replication-dependently expressed subtypes, and H1.0 and H1X, which are replication-independently expressed subtypes^42^. Although the amount of histone H1.2 around the *TERT* TSS in TAF-I KD cells was slightly higher than that in WT cells (Fig. 6E), this difference was not remarkable compared with that observed in active histone markers (Fig. 5A,B). Furthermore, the amount of histone H1.0, H1.5, and H1X on the *TERT* TSS was not significantly different among WT and TAF-I KD cells (Fig. 6F). These results suggest that flux of histone H1s on the *TERT* TSS may not be required for the transcriptional regulation of *TERT* by TAF-I.

### Transient expression of exogenous TAF-I in TAF-I KD cells partially rescued the *TERT* transcription and induced the local reduction of CpG methylation at CGI-1 of *TERT*

**Figure 7:**
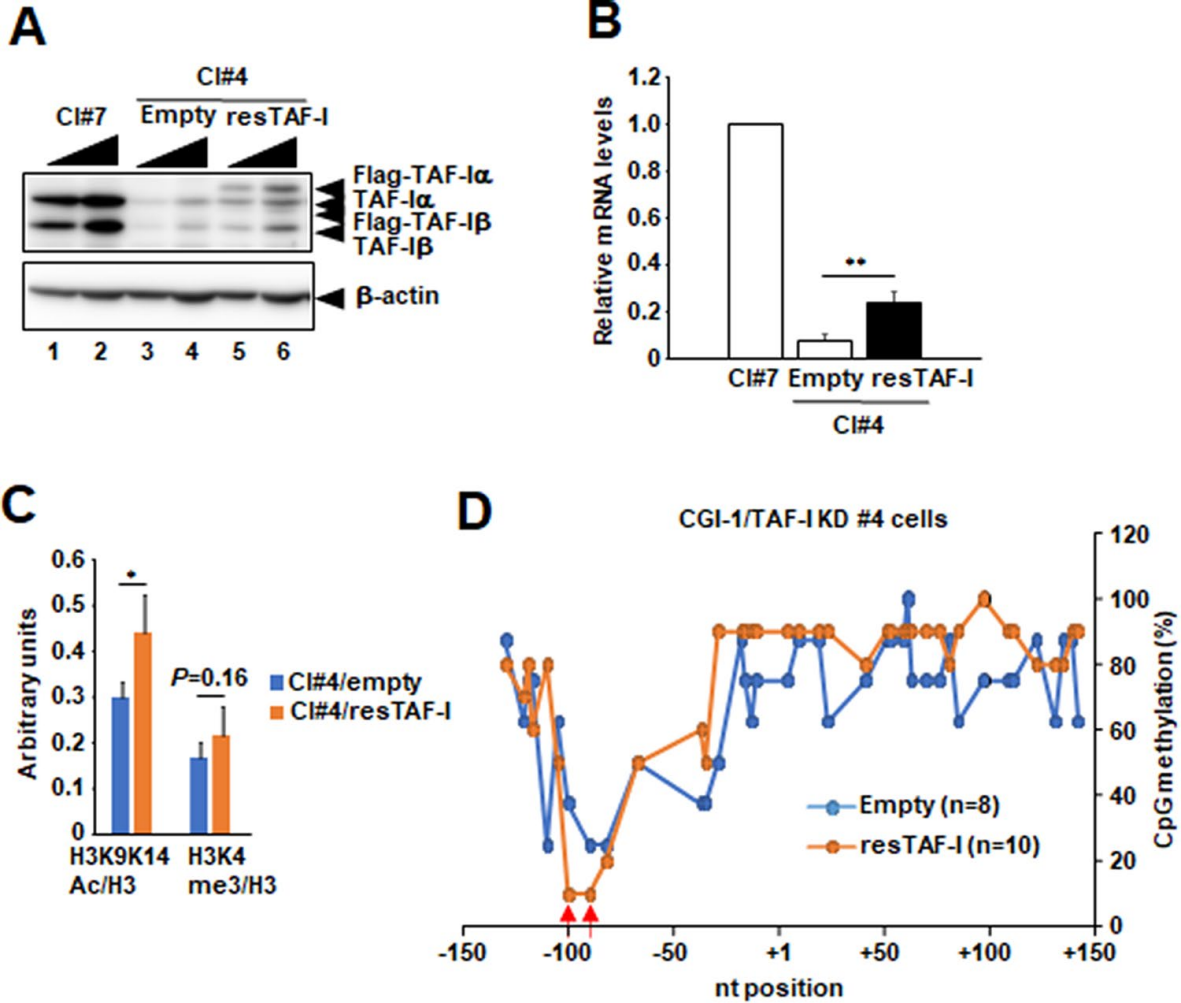
Transient expression of TAF-I partially rescued *TERT* transcription in TAF-I KD cells. (**A**) TAF-Iα and TAF-Iβ expression levels were examined by western blotting analyses in TAF-I KD clone #4 cells transfected with plasmid DNAs expressing shRNA-resistant Flag-TAF-Iα and Flag-TAF-Iβ (resTAF-I). Total cell extracts prepared from WT clone #7 cells (lanes 1–2), and TAF-I KD clone #4 cells transfected with empty vector DNA (lanes 3–4) or vector DNAs expressing resTAF-I (lane 5–6) were separated on 10% SDS-PAGE followed by western blotting analyses using anti-TAF-Iα/β and anti-β-actin antibodies. Ten (lanes 1, 3, and 5), and twenty (lanes 2, 4, and 6) µg of total protein prepared from each cell line was loaded. (**B**) The expression level of *TERT* mRNA was examined. Total RNA was isolated from each cell, and were subjected to Q-RT-PCR analyses to examine the expression level of *TERT* mRNA. The 18S rRNA was used for normalization as an internal control. Values represent the mean ± SD (n = 3). **P < 0.01 relative to #4 transfected with empty vector DNA. (**C**) The level of histone H3, H3K9K14ac, and H3K4me3 around the *TERT* TSS including core promoter was examined by ChIP assay. The value of each histone H3 modification was normalized by that of histone H3. Arbitrarily units were represented at vertical line. Values represent the mean ± SD (n = 3). *P < 0.01. (**D**) Bisulfite sequencing analyses. The methylation level of each CpG dinucleotide in CGI-1 of the *TERT* locus was compared between TAF-I KD clone #4 cells transfected with empty vector DNA and vector DNAs expressing resTAF-I. The horizontal line indicates nucleotide positions from the *TERT* TSS, and the vertical line indicates the percentage of CpG methylation. Two CpG nucleotides we observed reduction of methylation level by expression of resTAF-I were indicated by red arrows.

To get more insight about how TAF-I is involved in *TERT* transcription through the changes of epigenetic marks, we performed the rescue experiments by transiently expressing shRNA-resistant TAF-I (resTAF-I) in TAF-I KD cells. In our experimental condition, we just succeeded to express a few (less than 50% of endogenous TAF-I) amounts of resTAF-Iα and resTAF-Iβ in TAF-I KD cells (Fig. 7A, and Supplementary Fig. 1H–M). The amount of *TERT* mRNA in resTAF-I-expressed cells were approximately 3.5-fold increased compared to that in empty vector DNA-transfected cells, while its expression level does not reach that in WT #7 cells (Fig. 7B). We also found that the level of histone H3 K9K14ac was approximately 1.3-fold increased in resTAF-I-expressed cells compared to that in empty vector DNA-transfected cells, although the level of histone H3 K4me3 was not significantly increased (Fig. 7C). We also found that the CpG methylation level around nucleotide positions − 90 to − 100 at CGI-1 of *TERT* was reduced by transient expression of resTAF-I in TAF-I KD cells (Fig. 7D, indicated by red arrows). It is possible that TAF-I initially induces the reduction of CpG methylation around nucleotide positions − 90 to − 100 at CGI-1 and the increase of histone H3 acetylation around TSS to upregulate *TERT* transcription from the epigenetically transcription-repressed state.

### Down-regulation of *TERT* transcription by TAF-I KD was observed in not only HeLa cells but also other cancer cells

**Figure 8:**
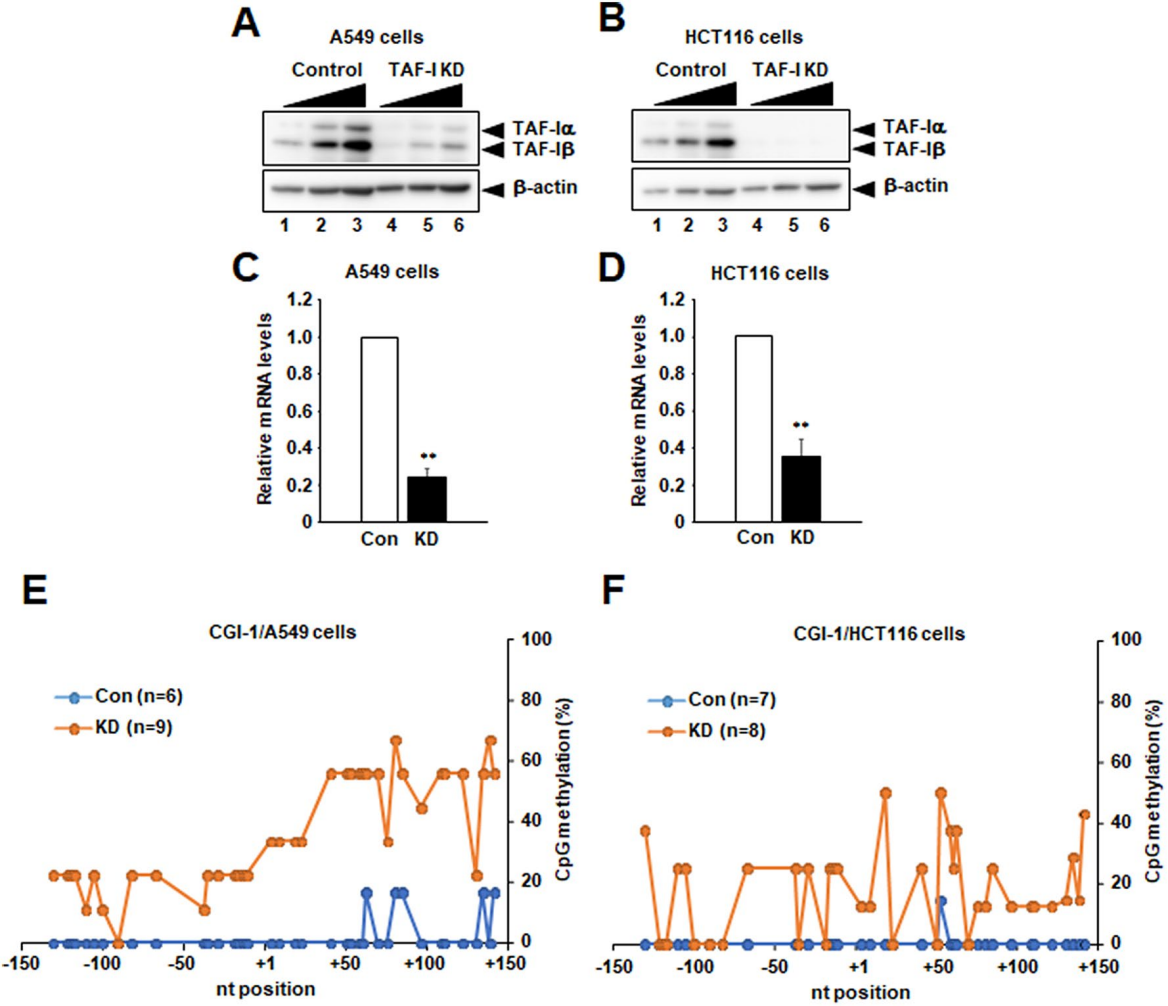
Downregulation of *TERT* transcription by TAF-I KD was observed in several cancer cells. (**A**,**B**) TAF-Iα and TAF-Iβ expression levels were examined by western blotting analyses. Total cell extracts prepared from control (lanes 1–3) and TAF-I KD (lanes 4–6) A549 cell lines (**A**), control (lanes 1–3) and TAF-I KD (lanes 4–6) HCT116 cell lines (**B**) were separated on 10% SDS-PAGE followed by western blotting analyses using anti-TAF-Iα/β and anti-β-actin antibodies. Five (lanes 1 and 4), ten (lanes 2 and 5) and twenty (lanes 3 and 6) µg of total protein prepared from each cell line was loaded. (**C**,**D**) The expression level of *TERT* mRNA was examined. Total RNA was isolated from control (Con) and TAF-I KD (KD) A549 cells (**C**) or HCT116 cells (**D**), and were subjected to Q-RT-PCR analyses to examine the expression level of *TERT* mRNA. The 18S rRNA was used for normalization as an internal control. Values represent the mean ± SD (n = 3). **P < 0.01 relative to respective control cells. (**E**,**F**) Bisulfite sequencing analyses. The methylation level of each CpG dinucleotide in CGI-1 of *TERT* was compared between control and TAF-I KD A549 cells (**E**) or HCT116 cells (**F**). The horizontal line indicates nucleotide positions from the *TERT* TSS, and the vertical line indicates the percentage of CpG methylation.

HeLa cells, an HPV-18-positive cervical cancer cell line, are well studied to clarify the regulatory mechanisms of *TERT* gene expression. Although we found that TAF-I is involved in transcriptional activation of *TERT* in HeLa cells, whether it is specific for HeLa cells or general for other cancer cells is unclear. To answer this question, we tried to establish TAF-I KD cell lines using other cancer cells. We succeeded to obtain stable TAF-I KD cell lines using A549 cells (a lung cancer) and HCT116 cells (a colorectal cancer). In TAF-I KD A549 cells and HCT116 cells, the expression level of TAF-Iα and TAF-Iβ were reduced to approximately 25% or less than 25% of that in control cells, respectively (Fig. 8A,B, and Supplementary Fig. 1N–R). In both TAF-I KD A549 and HCT116 cells, the amount of *TERT* mRNA was reduced to approximately 30% of that in control cells, suggesting that TAF-I is involved in the *TERT* transcription in not only HeLa cells but also A549 and HCT116 cells (Fig. 8C,D). To examine whether TAF-I is involved in the epigenetic changes around *TERT* TSS in A549 and HCT116 cells, we performed bisulfite sequencing analyses to evaluate the level of CpG methylation at CGI-1 of *TERT*. In contrast to HeLa cells, CpG dinucleotides at CGI-1 of *TERT* were almost entirely unmethylated in both A549 and HCT116 control cells (Fig. 8E,F). However, the level of CpG methylation at CGI-1 of *TERT* were entirely increased in both TAF-I KD A549 and HCT116 cells by TAF-I KD (Fig. 8E,F). Taken together, these results suggest that TAF-I is generally involved in *TERT* transcription through the alterations of CpG methylation around *TERT* TSS in several kinds of cancer cells.

## Discussion

In this study, we found that TAF-I is a novel regulator of telomere DNA synthesis through the transcriptional activation of *TERT* through epigenetic mechanism in human cancer cells. TAF-I maintains the histone H3 modifications involved in transcriptional activation and hypomethylated cytosines in CpG dinucleotides around the TSS including minimal core promoter in the *TERT*.

Because a variety of cancer cells acquire the reactivation of telomerase, the transcriptional activation of *TERT* is a crucial step for occurrence and recurrence of human cancer cells. It is well known that several nucleotide mutations in the *TERT* promoter such as C228T and C250T, both generating new binding sites for ETS family transcription factors, are frequently observed in human cancer cell lines. In addition to these genetic changes, epigenetic changes such as DNA methylation of CpG dinucleotides and histone modifications are also involved in the activation of *TERT* transcription. We found that TAF-I KD enhanced the methylation level of CpG dinucleotides around the TSS including minimal core promoter, but not the upstream region overlapping to THOR, in the *TERT* locus in HeLa cells. Furthermore, we observed that TAF-I KD simultaneously induces the reduction of *TERT* mRNA and upregulation of CpG methylation at CGI-1 of *TERT* in A549 and HCT116 cells. Therefore, it is possible that TAF-I is involved in the transcriptional activation of *TERT* through the maintenance of low CpG methylation level around TSS but not THOR in a variety of cancer cells. A previous report showed the dual-methylation pattern at the *TERT* promoter, in which the THOR is hypermethylated while the minimal promoter including C228T and C250T sites is hypomethylated in thyroid cancer cell lines^16^. Such dual-methylation pattern was also reported in other study^18^. These studies raise a possibility that the CpG methylation in the *TERT* locus is separately regulated at the THOR and the minimal promoter, and both regulations are important for the reactivation of *TERT* in cancer cells. Our findings become a clue to clarify how the locus-specific CpG methylation is regulated in the *TERT* locus. Interestingly, the methylation level at nucleotide position -67 from TSS, juxtaposed to the C228 nucleotide, showed the most marked difference between WT and TAF-I KD cells (WT is approximately 30%, and KD is approximately 95% in Fig. 4A,B, indicated in a red arrow). It is possible that the maintenance of low CpG dinucleotide methylation around C228 nucleotide is also important for the upregulation of the *TERT* transcription. In this study, we used HeLa cells, a human cervical cancer cell line, and observed neither C228T nor C250T mutations in genomic DNA, suggesting that these genetic changes do not occur and contribute to the *TERT* transcription in HeLa cells. However, it is possible that TAF-I-mediated maintenance of low CpG methylation cooperatively facilitates the reactivation of telomerase in combination with C228T mutation by assisting efficient binding of an ETS family transcription factor to the *TERT* promoter. Furthermore, we observed that transient expression of exogenous TAF-I partially rescues the *TERT* transcription in concert with the reduction of CpG methylation around nucleotide positions − 90 to − 100 at CGI-1 of *TERT* in TAF-I KD HeLa cells. Because the DNA around nucleotide positions − 90 to − 100 contains several Sp1 binding sites and also a C250 nucleotide (Fig. 4A), it is probable that this DNA region has an important role in *TERT* transcription. It is possible that TAF-I initially acts on DNA around nucleotide positions − 90 to − 100 at CGI-1 to change the *TERT* to the epigenetically transcription-activated state. We also observed that the levels of histone H3 K9K14ac and K4me3 are also reduced at the *TERT* TSS in TAF-I KD cells in along with increase of CpG methylation, although the changes of histone modification level seemed to be not completely correlated with that of *TERT* mRNA. It is likely that transcription of *TERT* is cooperatively regulated by many factors including histone modifications, DNA methylations, and transcription factors. We observed that not only the level of histone modifications but also the level of CpG methylation and promoter-bound Sp1 are also regulated by TAF-I KD. Thus, the level of histone modifications may be not simply correlated with the level of *TERT* transcription. These results suggest that TAF-I is involved in transcriptional activation of *TERT* through the maintenance of multiple epigenetic marks generally involved in the transcriptional activation. Sp1 may be one of key factors involved in TAF-I-dependent epigenetic changes around *TERT* TSS.

TAF-I is a multifunctional protein involved in many cellular processes including transcription^28,29^, DNA repair^46^, M-phase chromosome dynamics^47^, cell migration^48^, apoptosis^49^, and so on. As a clinical significance of *TAF-I*, *TAF-Iβ* but not *TAF-Iα* was identified as *SET* which consists of a part of translocated gene found in an acute leukemia patient^50^. Several reports suggested that expression level of TAF-I is increased and implicated in poor clinical outcomes in many types of cancer cells^51–56^. These reports suggest that TAF-I possibly has an oncogenic activity, however its detailed function in cancer progression is not completely understood. TAF-I suppresses the p53-mediated transcription through the inhibition of its acetylation like an INHAT^57^. Furthermore, it is reported that inhibition of PP2A by TAF-I is important for tumorigenesis in several cancer cells. In addition to these observations, this study proposes that TAF-I is involved in the occurrence and/or recurrence of cancer cells through the upregulation of *TERT* transcription as a novel epigenetic regulator. In this study, we did not clarify the detailed mechanisms how TAF-I specifically regulates epigenetic marks around the TSS in the *TERT* locus. It is reported that TAF-I functions as a transcriptional repressor in several genes by inhibiting acetylation of histone H3, although we observed the opposite effect on the *TERT*. It is possible that TAF-I subtilizes its functions of epigenetic regulation dependently on what genes are, where genomic loci are, and which type of cells are. It is difficult to directly identify which *cis*- and *trans*-acting factors cooperate with TAF-I in the *TERT* transcription due to its multifunctionality, but the methylome analyses to observe where genomic loci are affected by TAF-I may clarify the common pattern of gene structure under the control of TAF-I similar to the *TERT* locus.

## Materials and methods

### Cell cultures, antibodies, and chemicals

All WT and TAF-I KD HeLa cell lines were cloned by picking up well-isolated drug-resistant colonies using cloning rings^33^. TAF-I KD HeLa cell lines, HEK293T cells, T98G cells, A549 cells, and HCT116 cells were maintained in Dulbecco modified Eagle medium (DMEM, Nissui) containing 10% FBS. Antibodies used in this study were as follows: anti-TAF-Iα/β antibody (monoclonal antibody KM1720; Kirin-Kyowa Hakko), anti-β-actin, and anti-Sp1 antibodies from SIGMA; anti-histone H3, anti-histone H3 K4me3, anti-histone H3 K27me3, anti-pol II (8WG16), anti-histone H1.2, anti-histone H1.5, and anti-histone H1X antibodies from Abcam; anti-c-Myc antibody from CST; normal rabbit IgG, and anti-histone H3 K9K14ac antibodies from Millipore; anti-histone H1.0 antibody from ProteinTech Group, Inc. Actionomycin D was purchased from SIGMA. Decitabine was purchased from BioVision.

### Reverse transcription and quantitative PCR analyses

Total RNA was prepared from cells by acid guanidinium thiocyanate-phenol chloroform extraction (AGPC) method. The concentration of RNA in each sample was determined using a Nano Drop Lite spectrophotometer (Thermo Scientific). To analyze the level of the *TERT* mRNA and 18S rRNA, cDNA was synthesized from the total RNA prepared from HeLa cell lines using ReverTra Ace reverse transcriptase (TOYOBO) and a 9-mer random primer. The primer sets used for quantitative PCR (Q-PCR) were as follows: for *TERT*, 5′-CACGCGAAAACCTTCCTCAG-3′ and 5′-TGTTCCTCCCAGCCTTGAAG-3′; *TERC*, 5′-GCTGTTTTTCTCGCTGACTTTCA-3′ and 5′-GCAGCTGACATTTTTTGTTTGC-3′; *TET1*, 5′-TCCTGGTGCTATTCCAGTCC-3′ and 5′-CAGGAAGGAAGACAGGCAAG-3′; for 18S rRNA, 5′-AACGGCTACCACATCCAAGG-3′ and 5′-GGGAGTGGGTAATTTGCGC-3′. Q-PCR reactions were performed with FastStart SYBR Green Master (Roche) using Thermal Cycler Dice (TaKaRa). For the investigation of the *TERT* mRNA stability, 1 µM of actinomycin D was added to cell culture medium and incubated cells for 1, 2, 4 and 8 h. After collecting cells, total RNA was purified and subjected to reverse transcription and Q-PCR analyses as mentioned above.

### Telomere repeat amplification protocol (TRAP) assay and Q-TRAP assay

TRAP assay was basically performed as previously described^58^. Each cell line was collected by trypsinization and subjected to counting of cell numbers. Same number of each cell treated with ice-cold hypotonic buffer (10 mM Hepes–NaOH [pH7.5], 10 mM KCl, 1.5 mM MgCl_2_ and 1 mM DTT) was centrifuged, then supernatant was discarded. Cells further incubated with lysis buffer (10 mM Tris–HCl [pH7.4], 1 mM MgCl_2_, 1 mM EGTA, 0.5% CHAPS, 0.1 mM PMSF and 5 mM β-mercaptoethanol) on ice for 30 min were subjected to centrifugation, then supernatant was collected and stored in − 80 °C until use. Cell lysate prepared from desired number of cells was mixed with ACX primer, RNase inhibitor, KOD-Plus-PCR enzyme (TOYOBO), and with or without TS primer in PCR reaction buffer for KOD-Plus and incubated at 30 °C for 30 min, then subjected to PCR. When required, cell lysate was pre-incubated with RNaseA or heat-denatured at 95 °C for 30 min. PCR was performed with 26 cycles of 96 °C for 20 s and 60 °C for 1 min. PCR products were separated on 8% polyacrylamide gel electrophoresis in 1×TBE buffer, and subjected to EtBr staining. The primers used for TRAP assay were as follows: TS primer, 5′-AATCCGTCGAGCAGAGTT-3′; ACX primer, 5′-GCGCGGCTTACCCTTACCCTTACCCTAACC-3′. For Q-TRAP assay, same cell lysates used in TRAP assay were mixed with ACX primer, RNase inhibitor, TS primer, and KOD-Plus- SYBR mix (TOYOBO). Mixtures were incubated at 30 °C for 30 min, then directly subjected to Q-PCR analyses using Thermal Cycler Dice.

### Telomere-FISH assay

Telomere-FISH assay was basically performed as previously described with several modifications^34^. Each HeLa cell line grown on glass cover slip was washed with PBS (−) and fixed with 4% formaldehyde at room temperature for 10 min. Fixed cells on coverslip were briefly washed with 0.2×SSC, then dehydrated with 70, 85, and 90% EtOH in a stepwise fashion at room temperature for 3 min, respectively. After cells were incubated with 10 mM sodium citrate (pH6.5) at 85 °C for 10 min, cells were washed with PBS (−) and dipped through 25, 50 and 95% EtOH in a stepwise fashion at room temperature for 3 min, respectively. Cells were incubated with PBS (−) containing 0.2 mg/ml RNase A at 37 °C for 10 min, washed with PBS (−) and again dipped through 25, 50 and 95% EtOH in a stepwise fashion at room temperature for 3 min, respectively. Cells were incubated with hybridization buffer (10 mM Tris–HCl [pH7.4], 70% formamide, and 1xBlocking reagent [Roche]) containing 100 nM TelC-Cy3 PNA probe (Panagene) at 86 °C for 6 min, and further incubated at room temperature for 2.5 h in the dark. Cells were washed with hybridization buffer at room temperature for 15 min, followed by PBS (−) containing 0.05% Tween20 at room temperature for 5 min. Finally, DNA was stained with DAPI, then coverslips were sealed on slide glass and subjected to observation using LSM700 confocal microscope. Data analyses were performed by Image J Fiji software.

### Terminal restriction fragment (TRF) assay

Each collected HeLa cell line was treated with ProteinaseK, and whole nucleic acids were purified by phenol/CHCl_3_ extraction and EtOH precipitation. Whole nucleic acids were treated with RNaseA, then again subjected to phenol/CHCl_3_ extraction and EtOH purification to purify genomic DNA. After each genomic DNA was digested with HinfI and RsaI, DNA were purified by phenol/CHCl_3_ extraction and EtOH purification. Ten µg of each DNA was separated by 0.8% agarose gel electrophoresis in 1×TBE buffer at 100 V constant for 4.5 h, DNA was visualized by staining with GelRed, then transferred onto Biodyne nylon membrane (PALL) by alkali transfer method. After membrane was pre-hybridized with hybridization buffer (5×SSC, 5×Denhardt’s solution, 0.5 mM tetrasodium pyrophosphate, 10 mM Na_2_HPO_4_, 0.1% SDS, and 0.1 mg/ml sermon sperm DNA) at 37 °C for 1 h, ^32^P-labeled telomere probe was added and further incubated at 37 °C for overnight. After membrane was washed for three times with wash buffer (0.1×SSC and 0.1% SDS) at 22 °C for 7 min, images were taken by Typhoon FLA 7000. The telomere probe DNA was as follows: 5′-CCCTTACCCTTACCCTTA-3′. Telomere probe DNA was radiolabeled with [γ-^32^P]ATP by incubation with T4 polynucleotide kinase. The telomere length of each sample was evaluated using WALTER, an online toolset^59^.

### Cell growth assay

Cell growth was evaluated by WST-8 assay using CCK-8 reagents (DOJINDO). WT and TAF-I KD HeLa cells (5 × 10^3^ cells) were plated onto 96 well plates with 100 µL DMEM-10% FBS. After incubation for 24, 48, and 72 h, 100 µL of CCK-8 reagents was added to medium and incubated at 37 °C for 1 h. Absorbance at 450 nm was measured by microplate reader (TECAN).

### Plasmid transfection and luciferase assay

A series of *TERT* promoter-fused firefly luciferase reporter plasmid DNA (pTERT-Luc) was previously established^39^. HeLa cell lines were transfected with pTERT-Luc plasmid DNAs using GeneJuice^®^ (Novagen) in combination with a control plasmid DNA pEF1α-RLuc expressing *renilla* luciferase. At 48 h after transfection, cells were washed with PBS (−) and lysed in a cell lysis buffer for *renilla* luciferase (Promega) by three freezing–thawing cycles. The cell lysates and a firefly or *renilla* luciferase substrate (Promega) were mixed, and both luciferase activities were measured by Lumat LB9506 (BERTHOLD). The firefly luciferase activities were normalized by the *renilla* luciferase activity.

### Bisulfite sequencing analyses

Each collected HeLa, A549, and HCT116 cell line was treated with ProteinaseK, and whole nucleic acids were purified by phenol/CHCl_3_ extraction and EtOH precipitation. Whole nucleic acids were treated with RNaseA, then again subjected to phenol/CHCl_3_ extraction and EtOH purification to purify genomic DNA. Each DNA concentration was measured by Nano Drop mini. Two µg of each genomic DNA was incubated in 0.3 M NaOH at 37 °C for 15 min, then 1.9 M sodium bisulfite and 0.5 mM hydroquinone were added and further incubated in PCR machine with 15 cycles of 30 s at 95 °C and 15 min at 50 °C. DNAs were purified by EtOH precipitation, then subjected to PCR using TaKaRa EpiTaq™ HS DNA polymerase (TaKaRa). The primers used for PCR were as follows: for the CGI-1, 5′-CGGGATCCTGTTTTGTTTTTTTATTTTTTAG-3′ and 5′-CGGGATCCCCAACCCTAAAACCCC-3′; for the CGI-2, 5′-CGGGATCCTTGTTTTTAGGGTTTTTATATTATGGT-3′ and 5′-CGGGATCCCAAAACTAAAAAATAAAAAAACAAAAC-3′. PCR products were digested with BamHI and subcloned into BamHI-digested pcDNA3 plasmid DNA, then transformed into bacteria. Plasmid DNAs were isolated and subjected to sequencing reaction with BigDye terminator (ver3.1) using a primer corresponding to T7 promoter sequence.

### Chromatin immunoprecipitation (ChIP) assay

Each HeLa cell line was fixed with 1% formaldehyde by directly adding formalin into culture medium and incubated at room temperature for 10 min. After adding 0.125 M glycine and incubation at room temperature for 5 min to stop cross linking, cells were washed with PBS (−) and collected into tube by scraping and centrifugation. Cells were resuspended in lysis buffer (50 mM Tris–HCl [pH7.9], 10 mM EDTA, 1% SDS and 1 mM PMSF) and subjected to lyse by sonication. After centrifugation of cell suspensions, supernatants were collected and tenfold diluted with dilution buffer (16.7 mM Tris–HCl [pH7.9], 167 mM NaCl, 1.2 mM EDTA, 1.1% TritonX-100, 0.01% SDS and 1.1 mM PMSF). Protein A-Sepharose beads pre-incubated with BSA and sermon sperm DNA were added to each lysate and rotate at 4 °C for 6 h to pre-clear the lysate. Supernatants were collected by centrifugation, each antibody was added and incubated at 4 °C for overnight with rotation. Protein A-Sepharose beads were added to lysate and further incubated at 4 °C for 1 h. After beads were collected by centrifugation, washed with high salt wash buffer, LiCl wash buffer and TE in a stepwise fashion. DNA–protein complex bound with beads was eluted with elute buffer (0.1 M NaHCO_3_, 1% SDS and 10 mM DTT). Elutes and a part of lysate were incubated at 65 °C for 6 h to reverse cross-links, added with glycogen as a carrier, then DNAs were purified by phenol/CHCl_3_ extraction and EtOH precipitation. Q-PCR reactions were performed with KOD-Plus- SYBR mix (TOYOBO) using Thermal Cycler Dice (Takara). The value of DNA derived from lysate (input) was used for normalization to calculate the % of input. The primers used for PCR were as follows: for the *TERT* promoter TSS, 5′-AGCCCCTCCCCTTCCTTTCC-3′ and 5′-AGCGCACGGCTCGGCAGC-3′.

### Rescue experiments by expression of Flag-TAF-I in TAF-I KD HeLa cells

TAF-I KD HeLa clone #4 cells were transfected with pcDNA3.1(+)-Flag plasmid (empty vector DNA) or pcDNA3.1(+)-Flag-TAF-Iα and pcDNA3.1(+)-Flag-TAF-Iβ plasmids^28^ expressing short hairpin RNA-resistant TAF-Iα and TAF-Iβ using GeneJuice^®^ in combination with pBabe-puro plasmid expressing puromycin-resistant gene. At 24 h after transfection, 2 µg/mL puromycin was directly added to medium and cells were further incubated for 24 h. After medium including dead cells were removed from dishes, cells were rinsed with PBS (−), then fresh medium was added and further incubated for 48 h. Cells were harvested and subjected to western blotting, total RNA purification following Q-RT-PCR, ChIP assay, and bisulfite sequencing analyses.

### Establishment of stable TAF-I KD cell lines in A549 and HCT116 cells

A549 and HCT116 cells were transfected with pU6i-puro-shEGFP or pU6i-puro-shTAF-I plasmids^28^ using GeneJuice^®^. At 24 h after transfection, 2 µg/mL of puromycin was added to cell culture medium and further incubated for 24 to 36 h. After medium including dead cells were removed from dishes, cells were rinsed with PBS (−), fresh medium was added and further incubated for 12 h. Cells were once replated onto *ϕ*10 cm dishes with medium and maintain until cell colonies grow to appropriate size. Multiple well-isolated colonies were picked up by trypsinization using cloning rings, transferred into fresh medium, and maintained until acquiring enough numbers of cells. Western blotting analyses using anti-TAF-I antibody were performed with obtained each cell clone. One of cell lines transfected with pU6i-puro-shEGFP which maintain the expression level of TAF-I similar to that in parental A549 or HCT116 cells was defined as control cells. Cell lines in which expression level of TAF-I is efficiently reduced compared to that in control cells were defined as TAF-I KD cells.

## Supplementary Information

Supplementary Information.
