# Supplementary material for: Template activating factor-I epigenetically regulates the TERT transcription in human cancer cells
Source: Sci Rep. 2021 Sep 6;11:17726. doi: 10.1038/s41598-021-97009-9 (PMC8421516; doi:10.1038/s41598-021-97009-9)
Supplement: Supplementary file 1 — Supplementary Information. [file 41598_2021_97009_MOESM1_ESM.pdf]

Template activating factor-I epigenetically regulates the *TERT* transcription in human cancer cells

Authors: Kohsuke Kato[1,*], Atsushi Kawaguchi[1,2,3] and Kyosuke Nagata[1,*]

Authors' affiliation:

[1]Department of Infection Biology, Faculty of Medicine, University of Tsukuba, 1-1-1 Tennodai, Tsukuba 305-8575, JAPAN

[2]Transborder Medical Research Center, University of Tsukuba, Tsukuba, Japan

[3]Microbiology Research Center for Sustainability, University of Tsukuba, Tsukuba, Japan

[*]**Corresponding authors:**

[1]Department of Infection Biology, Faculty of Medicine, University of Tsukuba, 1-1-1 Tennodai, Tsukuba 305-8575, JAPAN

E-mail address: knagata@md.tsukuba.ac.jp (K. Nagata)

: kkato@md.tsukuba.ac.jp (K. Kato)

Supplementary figure 1 **K Kato. et al.**

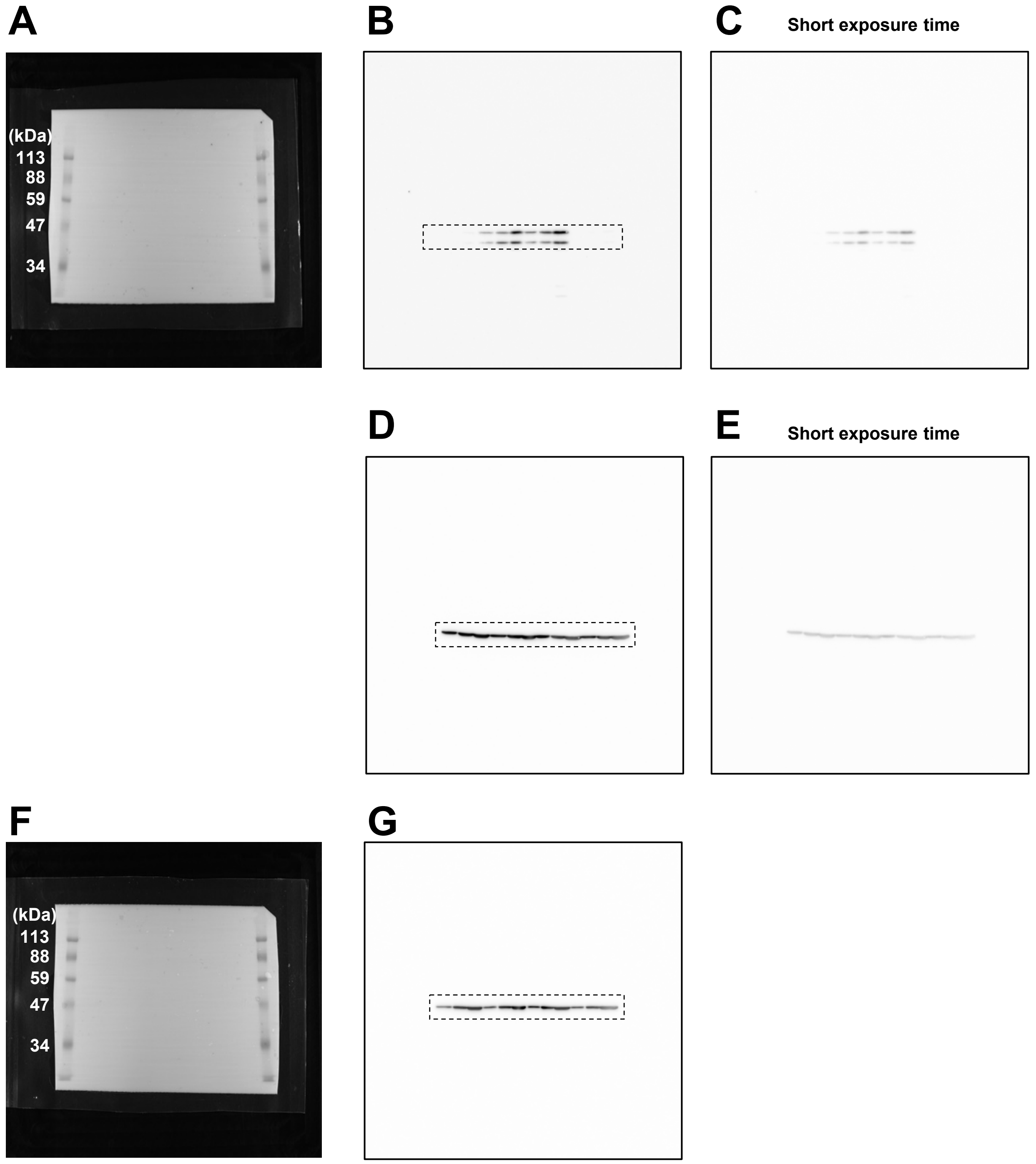

Supplementary figure 1 (continued) **K Kato. et al.**

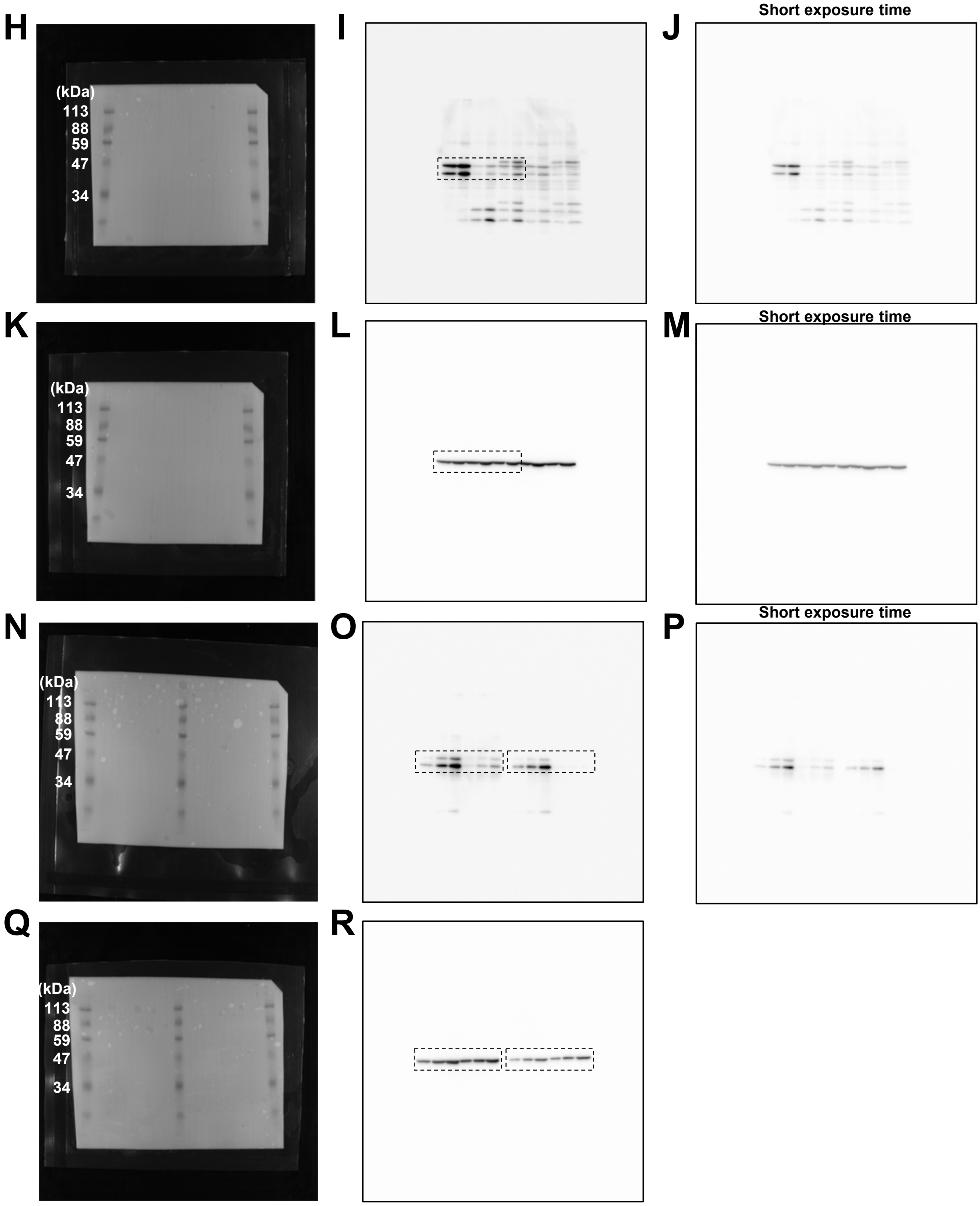

Supplementary figure 2 K Kato. et al.

A

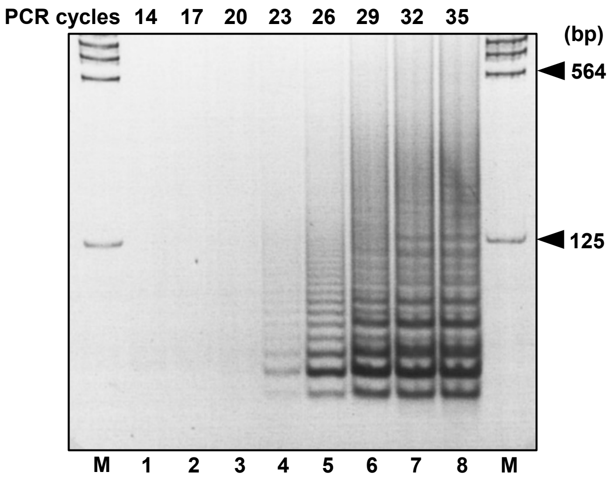

B

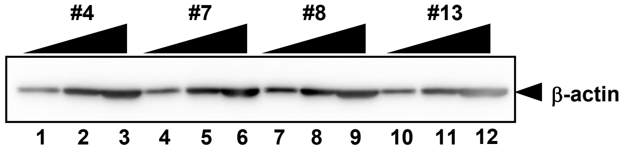

Supplementary figure 3

K Kato. et al.

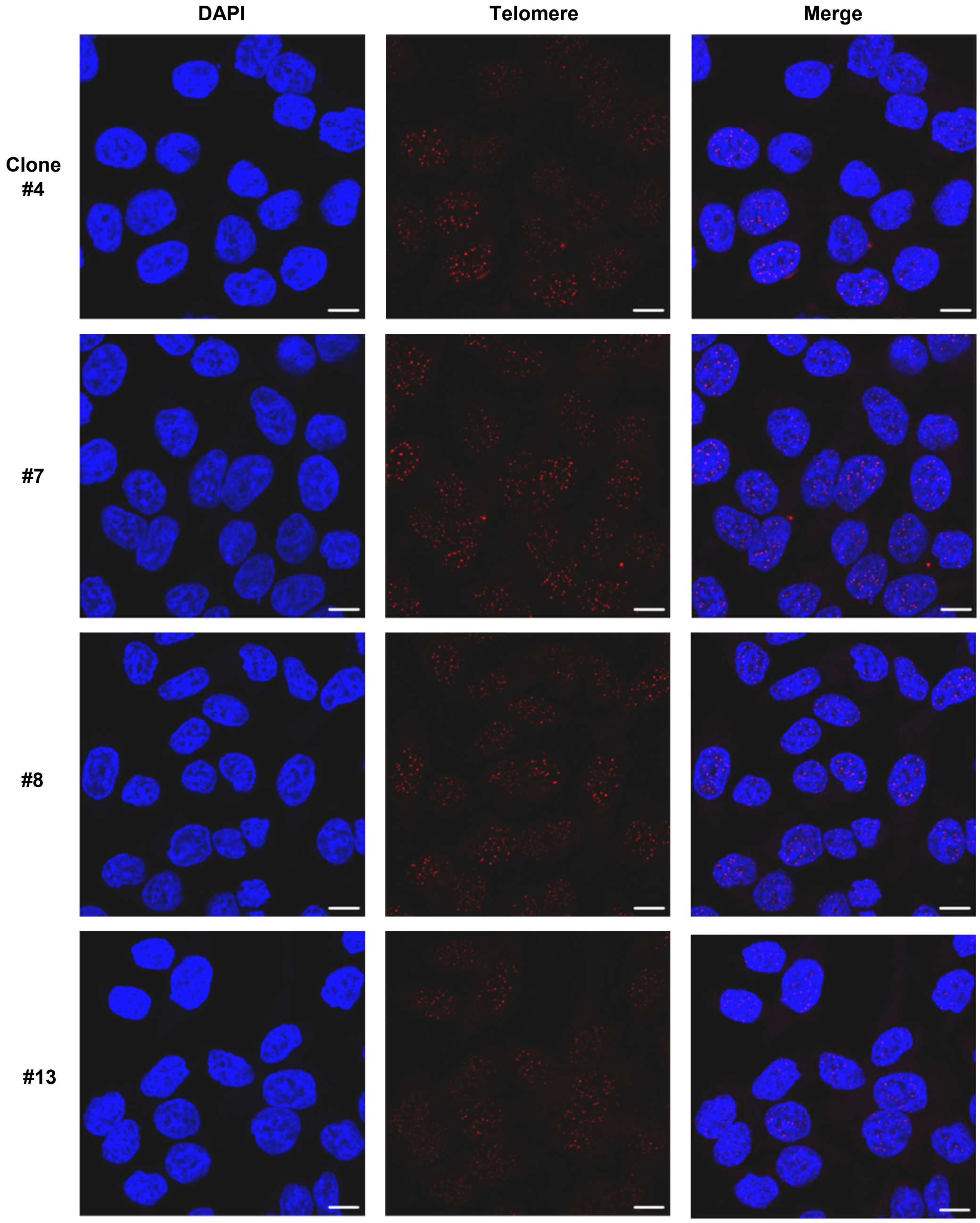

Supplementary figure 4

K Kato. et al.

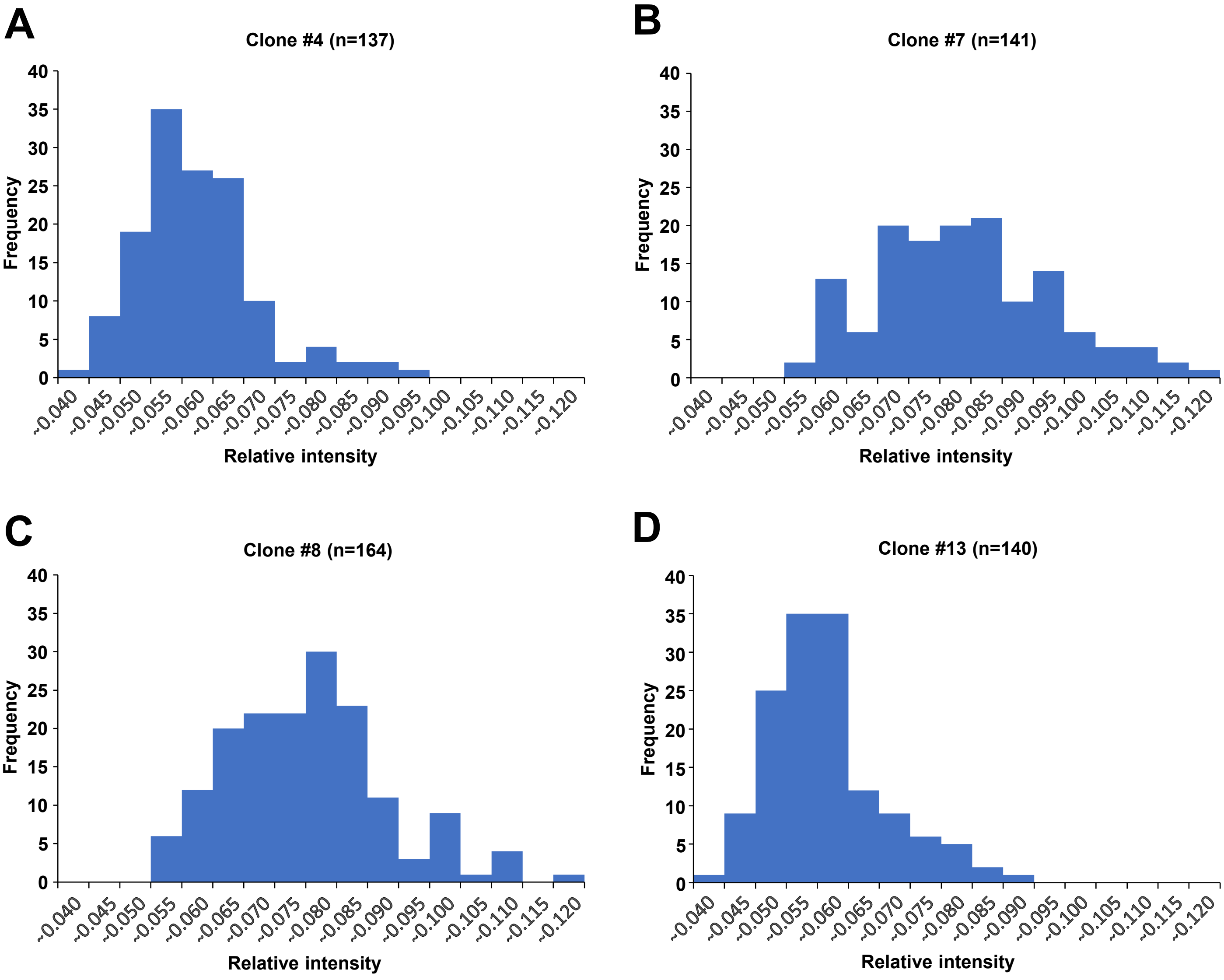

Supplementary figure 5

K Kato. et al.

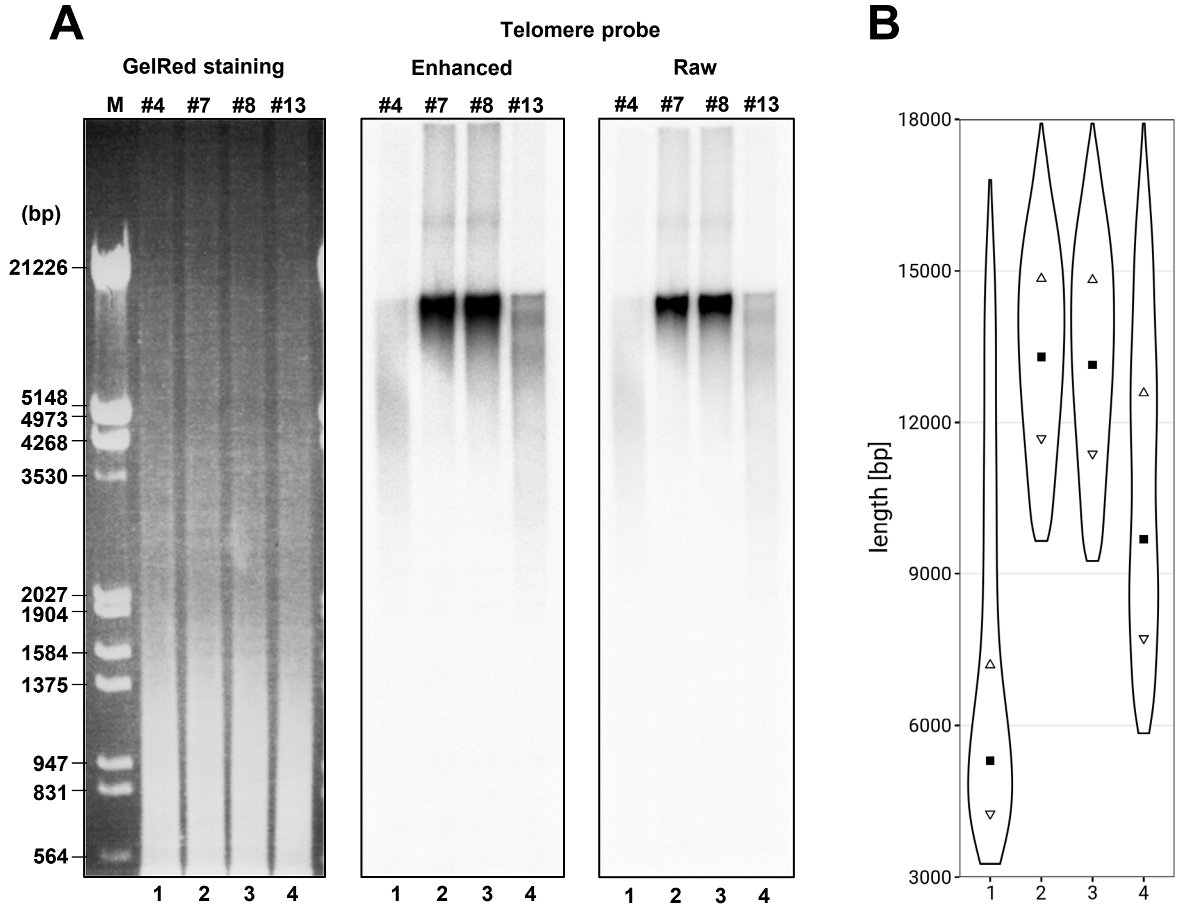

Supplementary figure 6

K Kato. et al.

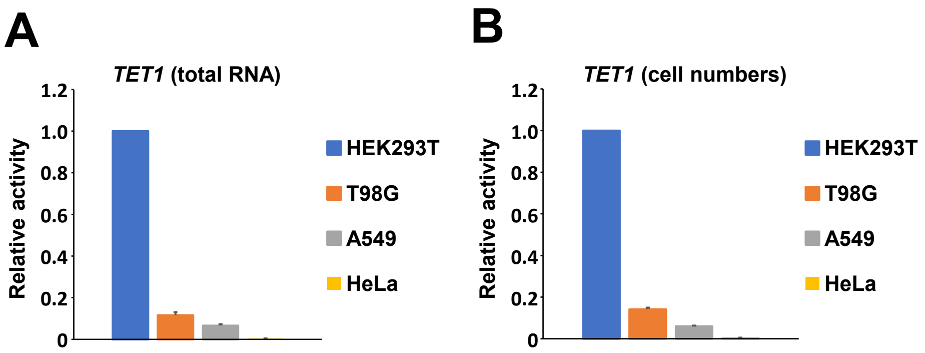

Supplementary figure 7 **K Kato. et al.**

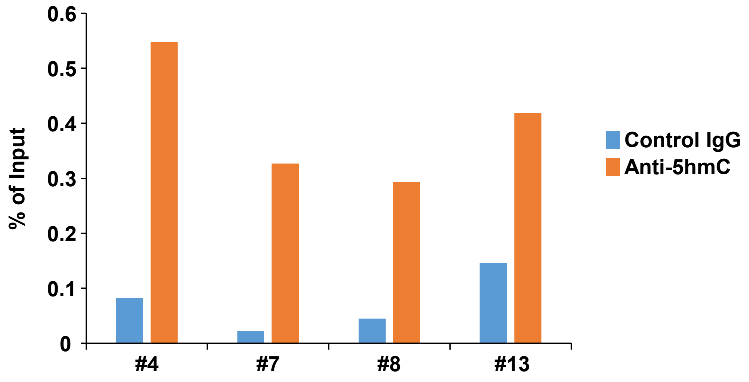

Supplementary figure 8 K Kato. et al.

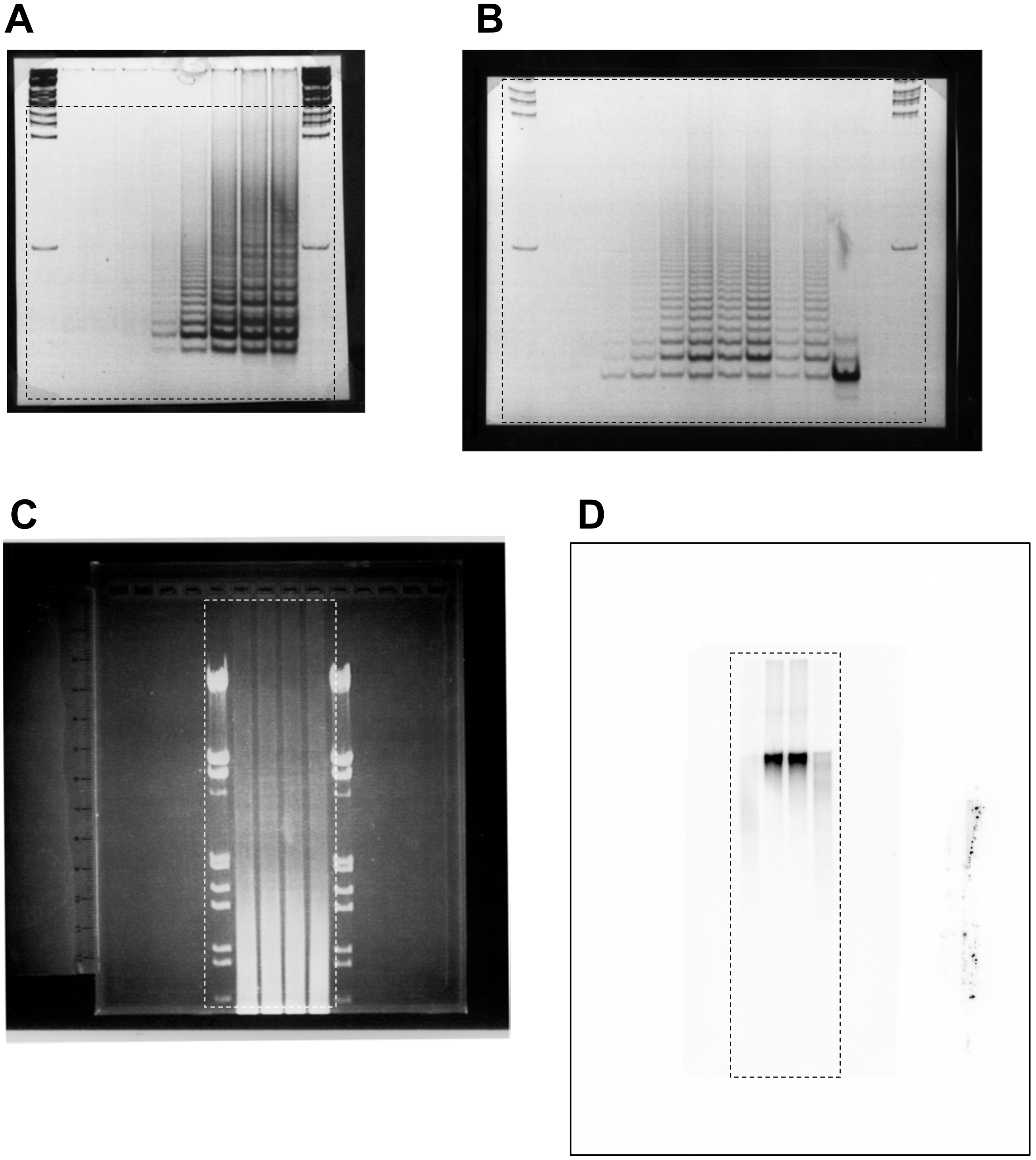

Supplementary information

Figure legends

Supplementary figure 1. Raw images for western blotting analyses.

The raw images of membrane (A), chemical luminescence signals corresponding to TAF-Iα and TAF-Iβ (B and C), and β-actin (D and E) in figure 1A were shown. Because a molecular weight of TAF-I is very similar to that of β-actin, after detection of TAF-I with anti-TAF-I antibody, same membrane was intensively washed for over-night and incubated with anti-β-actin antibody to detect β-actin. Where cropped blots are represented by box with dashed lines. The raw images of membrane (F), chemical luminescence signals corresponding to β-actin (G) in supplementary figure 2B were shown. Where cropped blots are represented by box with dashed lines. The raw images of membrane (H and K), chemical luminescence signals corresponding to TAF-Iα, TAF-Iβ, Flag-TAF-Iα, and Flag-TAF-Iβ (I and J), and β-actin (L and M) in figure 7A were shown, respectively. Where cropped blots are represented by box with dashed lines. The raw images of membrane (N and Q), chemical luminescence signals corresponding to TAF-Iα and TAF-Iβ (O and P), and β-actin (R) in figures 8A and 8B were shown, respectively. Where cropped blots are represented by box with dashed lines. Non-overexposed images of chemical luminescence among multiple images taken with different exposure times at the serial mode of Fusion Solo imaging system were represented. In parallel with raw images used for body figures by cropping desired area, other images taken with shorter exposure time were also shown except for panels (G) and (R) which taken by the shortest exposure time.

Supplementary figure 2. Several experiments for the establishment of TRAP assay.

(A) The determination of appropriate PCR cycles in TRAP assay. The extract derived from 2.5 x 10^3 cells of WT clone #7 cells was incubated with TS primer and ACX primer, RNase inhibitor, KOD-Plus- PCR enzyme in PCR reaction buffer for KOD-Plus at 30ºC for 30 min, then subjected to PCR. PCRs were performed with different cycles (14, 17, 20, 23, 26, 29, 32, and 35, respectively) of 96ºC for 20 sec and 60ºC for 1 min. PCR products were separated on 8% polyacrylamide gel electrophoresis in 1xTBE buffer, and subjected to EtBr staining. From this result, we used 26 PCR cycles for the analyses. (B) The efficiency of protein extraction

from cells for TRAP assay was examined by western blotting analyses. Each cell extract derived from 2.5 x 10^3 cells (lanes 1, 4, 7, and 10), 7.5 x 10^3 cells (lanes 2, 5, 8, and 11), and 2.5 x 10^4 cells (lanes 3, 6, 9, and 12) was loaded on 10% SDS-PAGE followed by western blotting analyses using an anti-β-actin antibody.

Supplementary figure 3. Representative images used for Telo-FISH analyses.

DAPI, Cy3 (telomere) and merged images of WT clones #7 and #8, TAF-I KD clones #4 and #13 HeLa cell lines in Telo-FISH analyses were shown. Scale bar represents 10 μm.

Supplementary figure 4. Distributions of cell numbers against relative telomere intensity was represented by Histograms.

Frequency (cell numbers) at each relative telomere intensity in HeLa cell lines clone #4 (A), #7 (B), #8 (C), and #13 (D) was represented as histograms. Images of DAPI staining and telomere-FISH were taken from cells, and each signal intensity was measured by ImageJ Fiji software. A value of total telomere intensity in one cell nucleus was normalized by dividing it with a value of total DAPI signal in same cell nucleus. The horizontal line indicates relative intensities, and the vertical line indicates the frequency.

Supplementary figure 5. TRF assay.

(A) Ten μg of *Hinf*I and *Rsa*I-treated genomic DNA purified from each HeLa cell line was separated on 0.8% agarose gel electrophoresis. The image stained with GelRed was shown in left panel. Lamda phage DNA cut with *EcoR*I and *Hind*III was loaded as DNA size marker. DNA was transferred onto nylon membrane, and subjected to southern blotting with ^{32}P-labeled telomere probe DNA. The enhanced and raw phospho-images were shown in center and right panels, respectively. (B) Telomere length of each TRF sample was quantified using WALTER toolset, then the results were represented by violin plot. Each number at the bottom corresponds to the lane number in gel and phospho-images. The rectangles indicate the weighted median, and the inverted triangles and triangles indicate the 25th and 75th percentiles, respectively. The shape of the violin plot describes the intensity changes between the minimum and maximum in analyzed area of TRFs.

Supplementary figure 6. The expression level of *TET1* mRNA was examined in several cancer cells. The expression level of *TET1* mRNA was examined by Q-RT-PCR analyses in HEK293T, T98G, A549, and HeLa cells. The 18S rRNA was used for normalization as an internal control. Acquired results when same amount of total RNA (A) or total RNA derived from same cell numbers (B) was subjected to Q-RT-PCR were represented. Values represent the mean ± SD (n=3).

Supplementary figure 7. MeD-IP assay to examine the level of 5hmC on the TSS of *TERT* Each genomic DNA was purified from WT and TAF-I KD HeLa cells by following a standard protocol. After 1 μg of each genomic DNA was denatured at 99ºC for 10 min, IP buffer was added to adjust to 10 mM Tris-HCl (pH7.4), 150 mM NaCl, and 0.05% TritonX-100. Rabbit control IgG or anti-5hmC antibody (MBL) was added and incubate at 4ºC for 2 hours, then Protein A-Sepharose beads were added and further incubated at 4ºC for 2 hours. Beads were collected by discarding supernatant after centrifugation, washed with IP buffer for 3 times, then DNAs were eluted with adding 1xProK buffer. DNAs were purified by phenol/$CHCl_3$ extraction and EtOH precipitation, then subjected to Q-PCR analyses using same primer set used in ChIP assay. Purified genomic DNA was also subjected to Q-PCR analyses as input samples. Data were represented as % of input.

Supplementary figure 8. Raw images of gels and southern blotting in TRAP and TRF assays. The raw images of DNA-derived fluorescent signals in gels (A) in supplementary fig. 2A, (B) in fig. 1D, and (C) in supplementary fig. 5A were shown, respectively. The raw phospho-image (D) in supplementary fig. 5A was also shown. Where cropped images are represented by box with dashed lines.
